# The essential roles of m^6^A RNA modification to stimulate ENO1-dependent glycolysis and tumorigenesis in lung adenocarcinoma

**DOI:** 10.1186/s13046-021-02200-5

**Published:** 2022-01-25

**Authors:** Lifang Ma, Xiangfei Xue, Xiao Zhang, Keke Yu, Xin Xu, Xiaoting Tian, Yayou Miao, Fanyu Meng, Xiaoxin Liu, Susu Guo, Shiyu Qiu, Yikun Wang, Jiangtao Cui, Wanxin Guo, You Li, Jinjing Xia, Yongchun Yu, Jiayi Wang

**Affiliations:** 1 Department of Clinical Laboratory Medicine, Shanghai Chest Hospital, Shanghai Jiao Tong University, No. 241 West Huaihai Road, 200030 Shanghai, China; 2 Shanghai Institute of Thoracic Oncology, Shanghai Chest Hospital, Shanghai Jiao Tong University, No. 241 West Huaihai Road, 200030 Shanghai, China; 3 Department of Clinical Laboratory Medicine, Shanghai Tenth People’s Hospital of Tongji University, 200072 Shanghai, China; 4 Department of Bio-bank, Shanghai Chest Hospital, Shanghai Jiao Tong University, 200030 Shanghai, China; 5 Nursing Department, Shanghai Chest Hospital, Shanghai Jiao Tong University, 200030 Shanghai, China; 6 Department of Respiratory Medicine, Shanghai Chest Hospital, Shanghai Jiao Tong University, No. 241 West Huaihai Road, 200030 Shanghai, China

**Keywords:** METTL3, ALKBH5, YTHDF1, translation, lung cancer, RNA-protein interaction

## Abstract

**Background:**

Lung adenocarcinoma (LUAD)  is the most common subtype of lung cancer. Patient prognosis is poor, and the existing therapeutic strategies for LUAD are far from satisfactory. Recently, targeting N6-methyladenosine (m^6^A) modification of RNA has been suggested as a potential strategy to impede tumor progression. However, the roles of m^6^A modification in LUAD tumorigenesis is unknown.

**Methods:**

Global m^6^A levels and expressions of m^6^A writers, erasers and readers were evaluated by RNA methylation assay, dot blot, immunoblotting, immunohistochemistry and ELISA in human LUAD, mouse models and cell lines. Cell viability, 3D-spheroid generation, *in vivo* LUAD formation, experiments in cell- and patient-derived xenograft mice and survival analysis were conducted to explore the impact of m^6^A on LUAD. The RNA-protein interactions, translation, putative m^6^A sites and glycolysis were explored in the investigation of the mechanism underlying how m^6^A stimulates tumorigenesis.

**Results:**

The elevation of global m^6^A level in most human LUAD specimens resulted from the combined upregulation of m^6^A writer methyltransferase 3 (METTL3) and downregulation of eraser alkB homolog 5 (ALKBH5). Elevated global m^6^A level was associated with a poor overall survival in LUAD patients. Reducing m^6^A levels by knocking out METTL3 and overexpressing ALKBH5 suppressed 3D-spheroid generation in LUAD cells and intra-pulmonary tumor formation in mice. Mechanistically, m^6^A-dependent stimulation of glycolysis and tumorigenesis occurred via enolase 1 (ENO1). *ENO1* mRNA was m^6^A methylated at 359 A, which facilitated it’s binding with the m^6^A reader YTH N6-methyladenosine RNA binding protein 1 (YTHDF1) and resulted in enhanced translation of ENO1. ENO1 positively correlated with METTL3 and global m^6^A levels, and negatively correlated with ALKBH5 in human LUAD. In addition, m^6^A-dependent elevation of ENO1 was associated with LUAD progression. In preclinical models, tumors with a higher global m^6^A level showed a more sensitive response to the inhibition of pan-methylation, glycolysis and ENO activity in LUAD.

**Conclusions:**

The m^6^A-dependent stimulation of glycolysis and tumorigenesis in LUAD is at least partially orchestrated by the upregulation of METTL3, downregulation of ALKBH5, and stimulation of YTHDF1-mediated ENO1 translation. Blocking this mechanism may represent a potential treatment strategy for m^6^A-dependent LUAD.

**Supplementary Information:**

The online version contains supplementary material available at 10.1186/s13046-021-02200-5.

## Background

Lung cancer is the first leading cause of cancer-related death worldwide [1], Non-small cell lung cancer (NSCLC) is the major histopathology subtype of lung cancer [2] and lung adenocarcinoma (LUAD) comprises approximately 60% of all NSCLC cases [3]. The prognosis of patients with LUAD, especially those at advanced stages, is poor. Ineffective therapeutic strategies and drug resistance are the main factors that contribute to tumor progression and the poor prognosis of LUAD patients. Therefore, better understanding of the mechanisms underlying LUAD progression are critical to identify potential new therapeutic targets for LUAD patients.

The m^6^A methylation is the most prevalent post-transcriptional modification within eukaryotic RNA [4]. It’s exciting that targeting m^6^A modification is considered as a promising way to impede tumor progression [5]. The processes of m^6^A methylation are dynamic and regulated by “writers”, “erasers” and “readers”, the so-called WER system. Writers and erasers oppositely catalyze m^6^A methylation, because they are methyltransferases and demethylases, respectively, and together coordinate m^6^A methylation status [6, 7]. While writers and erasers are considered as the regulators of m^6^A modification, readers are the terminal effectors of m^6^A reasoning that these enzymes recognize and guide m^6^A methylated RNAs for final outcome [8, 9]. METTL3, methyltransferase 14 (METTL14) and WT1 associated protein (WTAP) are the core components of the writer complex, while ALKBH5 and FTO alpha-ketoglutarate dependent dioxygenase (FTO) are responsible for the function as of erasers [10, 11]. Members of the YTH and insulin like growth factor 2 mRNA binding protein (IGF2BP) families and heterogeneous nuclear ribonucleoprotein A2/B1 (hnRNPA2B1) function as readers [12, 13]. Emerging studies have demonstrated that m^6^A modification and the WER system are critical for tumor initiation and progression [14, 15]; however, whether and how the coordination of WER systems influences m^6^A-dependent LUAD tumorigenesis is currently incompletely known.

Increased glycolysis, which involves elevated glucose uptake and lactate production is a hallmark of cancer metabolism [16]. Elevation glycolysis is regarded as an adaptation of cancer cells to the hypoxic microenvironment, thus providing continuous energy to promote cell proliferation, invasion and migration [17, 18]. The enolase (ENO) family, which catalyzes the generation of phosphoenolpyruvate (PEP) from 2-phospho-d-glycerate (2-PGA), is the key player that increases the global levels of glycolysis [19]. Targeting glycolysis has been explored as a therapeutic approach for cancer [20]. Emerging studies have revealed that m^6^A modification is associated with tumor initiation and progression, and m^6^A-dependent glycolysis is important for tumor progression in colorectal and gastric cancers [14, 21, 22]. However, the regulation of m^6^A and its relationship to glycolysis in LUAD remains unknown.

In this study, the regulations and functions of m^6^A modification were investigated via cell-based experiments, mice and three independent cohorts of human LUAD. The effects of m^6^A and the involved m^6^A readers were also investigated. We uncovered that ENO1-dependent glycolysis is critical for m^6^A to stimulate LUAD progression, and this effect is mediated via the m^6^A reader YTHDF1. In preclinical mice models, new treatment strategies against high m^6^A-level LUAD were also explored in the present study.

## Materials and methods

### Human tissue samples

Clinical tissue specimens of cohort#1 and #3 were obtained from Shanghai Chest Hospital (Shanghai, China) and tissue microarray slides loaded with LUAD from cohort#2 were purchased from Shanghai OUTDO Biotech LTD (Shanghai, China). All the basal information of patients is provided in Supplementary Tables 1, 2 and 3. Written informed consents were obtained from each patient, and the study was approved by the ethics and research committees of the Shanghai Chest Hospital.

### Cell culture, reagents and plasmids

The human lung epithelial cell line BEAS-2B, human bronchial epithelial cell line 16HBE, human LUAD cell lines A549, NCI-H1299, PC-9, NCI-H1975, NCI-H441, NCI-H1650, HCC827, NCI-H292, NCI-H2030, A427 and Calu-1, and the Murine Lewis lung cancer cell (LLC) line were obtained as described in our previous studies [23, 24]. All cells were routinely cultured in DMEM (Gibco, Carlsbad, CA, USA) supplemented with 10% FBS (HyClone, Logan, UT, USA) and 1% penicillin-streptomycin (Invitrogen, Carlsbad, CA, USA). The reagents used in this study were as follows: cycloheximide (CHX, #C7698, Sigma, St Louis, MO, USA), ActinomycinD (ActD, #HY17559, MedChemExpress, Monmouth, NJ, USA) and 3-deazaadenosine (DAA, #S0787, Selleck, Houston, TX, USA). For CRISPR-Cas9 knockout of *ALKBH5*, FTO, *METTL3*, *ENO1* and *YTHDF1*, single guide RNAs (sgRNAs) were cloned into the LentiCrisprV2 plasmid. Lentiviral-based METTL3, METTL14, WTAP, ALKBH5 and YTHDF1 expressing plasmids were purchased from GeneCopoeia Biotech (Rockville, MD, USA). Plasmids expressing sgRNA-resistant wild-type ENO1 or 359 A mutatnt (ENO1^WT^ and ENO1^Mut^, respectively) were generated by Zuorun Biotech (Shanghai, China). Sequences encoding wild-type YTHDF1 or YTHDF1 lacking the YTH-domain were cloned into the pCDNA3.1(+)-HA vectors. The sequences for sgRNA and primers are listed in Supplementary Tables 4.

### Quantitative real-time PCR (qPCR),12909 Immunoblotting (IB), Immunofluorescence (IF), Immunohistochemistry (IHC) and Enzyme-liked Immunsorbent assay (ELISA)

For qPCR, total RNA was extracted using TRIzol reagent (Invitrogen) and cDNA was synthesized using HiScript III RT SuperMix (Vazyme, Nanjing, China). The reaction was performed using the universal SYBR qPCR Master Mix (Vazyme). IB, IF and IHC were performed following conventional protocols. IHC scores were calculated as we described previously [24]. Besides IB, METTL3, ALKBH5, METTL14, WTAP, YTHDF1, ENO1 and FTO were also measured using ELISA kits from Lichen Biotech (Shanghai, China). The qPCR primer sequences and antibody information for IB, IF and IHC are listed in Supplementary Tables 5-6.

### Analysis of translation efficiency

The translation efficiency of endogenous ENO1 was calculated as the ratios between the levels of ENO1 protein and *ENO1* mRNA. The translation efficiency of the exogenous ENO1-LUC was calculated as the ratios between luciferase activity that measured from a reporter using the dual luciferase reporter gene assay kit (Promega, Madison, WI, USA) and the levels of *ENO1-LUC* mRNA. For the construction of the reporter, the partial *ENO1* open reading frame (ORF) sequences with or without mutation at 359 A were cloned upstream of luciferase-coding region in the pmir-GLO-based plasmids (Zuorun Biotech).

### Cell invasion, cell viability and 3D-spheroid formation assays

Transwell assay was performed to determine cell invasion ability following the conventional protocols. Cell viability was measured using the CellTilter-Glo cell viability assay kit (Promega, #G9682) in accordance with the manufacturer’s instructions. The 3D-spheroids were generated and cultured as we described previously [23, 24].

### Measurement of global m^6^A

The global m6A measured in the present study is specific to mRNA. Firstly, total mRNA was purified using the Dynabeads mRNA purification kit (Invitrogen). Global mRNA m^6^A levels were measured by the EpiQuick m^6^A RNA methylation assay kit (Abcam, #ab185912, Cambridge, MA, USA). For dot blot assays, mRNA was denatured and spotted on Biodyne ® Nylon Transfer Membranes followed by crossing-linked using UVP for 10 min. The anti-m^6^A antibody (Synaptic Systems, #202,003, Goettingen, Germany) was finally used to measure m^6^A levels.

### Photoactivatable ribonucleoside-enhanced crosslinking and immunoprecipitation (PAR-CLIP), RNA Immunoprecipitation (RIP) and RNA pull-down assay

PAR-CLIP and RIP experiments were performed as we previously described [23, 24]. The antibodies were as follows: anti-HA (Abcam, ab#1424), anti-m^6^A (Synaptic Systems, #202,003), anti-YTHDF1 (Abcam, #ab220162) and control IgG (Abcam, ab#172,730). The qPCR assay was performed to detect *ENO1* mRNA and the data were normalized to the input. The primers are listed in Supplementary Table 5.

For RNA pull-down assays, biotin-labeled partial *ENO1* ORF with or without artificial m^6^A modification at 359 A were synthesized by Takara (Dalian, China). Biotin-labeled RNA was incubated with cell lysates at 4 °C overnight. Streptavidin magnetic beads (Life Technologies, Carlsbad, CA, USA) were added to the reaction for 1 h. After washing, the enriched proteins were subjected into IB analysis.

### Polysome profiling

Cells were incubated with CHX (10 µg/ml) at 37 °C for 15 min. The cells were then lysed and subjected into 10-50% sucrose-gradient centrifugation for fractionation. Total RNA was finally extracted using TRIzol reagent and subjected into qPCR analysis. The primers used for detecting RNA are listed in Supplementary Table 5.

### Measurements of the decay of mRNA and protein

To evaluate the half-lives of mRNA and protein, cells were treated with ActD (5 µg/ml) or CHX (10 µg/ml) for indicated time. The expressions of ENO1 mRNA and protein were then determined by qPCR and IB assay, respectively.

### Measurement of glycolysis

The levels of ENO1 activity, lactate production, pyruvate, ATP and PEP were measured using kits which were purchased from Biovision (Milpitas, CA, USA) according to the manufacturer’s instructions. Glucose uptake was measured using glucose analog 2-NBDG (Selleck, #S8914). Extracellular acidification rate assay (ECAR) and oxygen consumption rate (OCR) were analyzed using the extracellular flux analyzer XF96 (Seahorse Bioscience, Billerica, MA, USA) with the glycolysis stress test kit (Agilent, # 103020-100Wilmington, DE, USA) and mitochondrial stress test kit (Agilent, # 103015-100), respectively.

### Mouse experiments

The conditional Cre-driven *Mettl3* knockout (*Mettl3*^*−/−*^) mice were obtained from Cyagen (Santa Clara, CA, USA). The Cre-driven *Kras*^*G12D/+*^; *p53*^*R172H/+*^ (*KP*) mice were obtained as descried in our previous study [24]. *KP* and *Mettl3*^*−/−*^ mice were bred to generate *KPM*^*−/−*^ mice. Afterwards, the *KP* and *KPM*^*−/−*^ mice were intranasally infected under anesthesia with adeno-associated virus type 5 (AAV5) expressing Cre to initiate lung tumorigenesis along with ALKBH5-expressing AAV5 or Empty AAV5 to generate *KPE*, *KPA*, *KPEM*^*−/−*^ and *KPAM*^*−/−*^ spontaneous LUAD mouse models. For generation of LLC-based intra-pulmonary tumor mouse models, 1 × 10^7^ LLC cells were injected into C57BL/6 mice via the tail vein. The numbers of intra-pulmonary tumor foci were counted after dissection.

For cell-derived xenograft (CDX) mouse models, 1.0 × 10^7^ H1299 or 1.5 × 10^7^ H1975 cells were subcutaneously injected into 4-6-week-old athymic nude mice. The tumors were monitored at indicated time points and isolated for further analysis after sacrifice. For patient-derived xenograft (PDX) mouse models, were constructed similar as described in our previous studies [23, 24].

After CDX^H1299^ and PDX xenografts were generated and reached similar sizes, DMSO, DAA (50 mg/kg), 2-Deoxy-D-glucose (2DG, Selleck, #S4701, 1000 mg/kg) or ENOblock (Selleck, #S7443, 20 mg/kg) were administrated every other day. For the experiments in Fig. 7O, *KPE* mice were administrated with DMSO, DAA (25 mg/kg), 2DG (500 mg/kg) or ENOblock (10 mg/kg) once a week for 2 months. All mouse experiments were approved by the institutional ethics committee of Shanghai Chest Hospital.

### Proteomics and bioinformatics

Proteomics were performed by Oebiotech LTD (Shanghai, China) to identify differential protein expression profiling. The data from Kaplan-Meier plotter database (http://www.kmplot.com) was extracted for analyzing survival information of LUAD with different expression levels of ENO1.

### Data availability

The raw data of proteomics have been deposited to the ProteomeXchange Consortium via the iProX partner repository with the data set identifier PXD027632 and PXD027633.

### Statistical analysis

Student’s t-test, one-way ANOVA, two-way ANOVA, *Chi*-square tests, Pearson analysis, Spearman rank-correlation analysis and log-rank tests were used to perform statistical analysis. The results are presented as mean ± SEMs from three independent experiments or indicated samples. *p<0.05, **p<0.01 were considered statistically significant and N.S. indicates no significance.

## Results

### M^6^A levels in LUAD are determined by METTL3 and ALKBH5

**Fig. 1:**
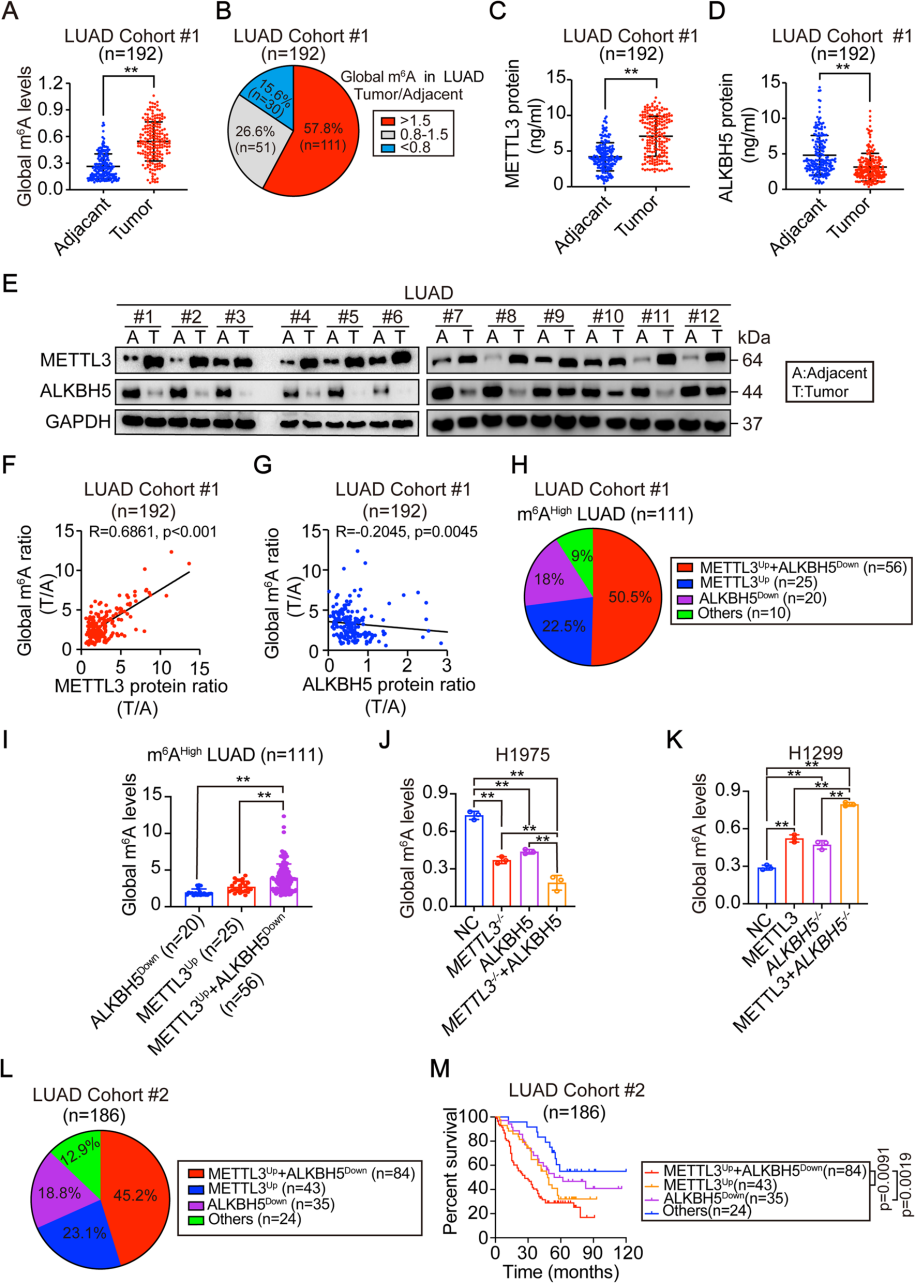
Global m^6^A was modulated by METTL3 and ALKBH5 in LUAD.(**A**) Global m^6^A levels were measured by m^6^A methylation assay in tumor and matched-adjacent tissues from LUAD patients. (**B**) The percentage of LUAD in cohort #1 with distinct tumor/adjacent ratio of global m^6^A, as indicated. (**C**-**D**) METTL3 (**C**) and ALKBH5 (**D**) protein levels in tumor and matched-adjacent tissues from LUAD patients, as measured by ELISA. (**E**) IB of METTL3 and ALKBH5 in tumor and matched-adjacent tissues from 12 LUAD patients. (**F**-**G**) Correlation between global m^6^A and METTL3 (**F**), and between global m^6^A and ALKBH5 (**G**) in LUAD patients. The global m^6^A and protein levels were calculated as the ratios between tumor and matched-adjacent tissues. (**H**) The percentage of cases with different METTL3 and ALKBH5 expressions, as indicated, in LUAD with high global m^6^A levels. (**I**) The global m^6^A levels in different groups with indicated METTL3 and ALKBH5 expression from LUAD with high global m^6^A levels. (**J**) Global m^6^A levels in control and H1975 cells with separate or combined METTL3 knockout and ALKBH5 overexpression. (**K**) Global m^6^A levels in control and H1299 cells with separate or combined METTL3 overexpression and ALKBH5 knockout. (**L**-**M**) Percentage (**L**) and overall survival (M) of LUAD patients with different METTL3 and ALKBH5 expression, as indicated in cohort #2. Statistical analysis was performed using t-test (**A**, **C**, **D**), spearman rank-correlation analysis (**F**, **G**), one-way ANOVA (**I**-**K**) and log-rank test (**M**). Data are presented as means ± SEMs from indicated samples or three independent experiments. **p < 0.01, indicates statistical significance

To manifest the roles of m^6^A in LUAD, global m^6^A levels were examined in matched adjacent-tumor tissues from LUAD patients. In cohort #1(n=192), global m^6^A levels were upregulated in LUAD compared with levels in matched adjacent tissues (Fig. 1 A). The ratio of m^6^A levels in tumor/adjacent tissues was more than 1.5 in 57.8% (111/192) of LUAD patients, whereas only 15.6% (30/192) of patients showed a ratio of less than 0.8 (Fig. 1B), suggesting that the elevation of m^6^A level is quite common in LUAD tumors.

Global m^6^A levels are determined by writers and erasers. Among the three major components of the writer complex, only METTL3 was upregulated in LUAD as determined by ELISA (Fig. 1 C); METTL14 and WTAP were expressed at similar levels in adjacent and tumor tissues (Supplementary Fig. 1 A-B). For the two major erasers, i.e. ALKBH5 and FTO, only ALKBH5 was downregulated in LUAD (Fig. 1D and Supplementary Fig. 1 C). To validate the ELISA results, we examined protein expressions in randomly chosen LUAD specimens using IB and IHC. The results were consistent with the ELISA results. METTL3 was upregulated while ALKBH5 was downregulated in tumors compared with levels in adjacent tissues in all the tested specimens (Fig. 1E and Supplementary Fig. 1D). Then, further found that global m^6^A levels were positively correlated with METTL3 and negatively correlated with ALKBH5 in LUAD (Fig. 1 F-G). In contrast, global m^6^A levels were not well correlated with METTL14, WTAP and FTO (Supplementary Fig. 1E-G). We next examined METTL3 and ALKBH5 expressions in the specimens with a tumor/adjacent tissue global m^6^A ratio of more than 1.5 (Fig. 1B). We found that 50.5% (56/111) of samples demonstrated both upregulated-METTL3 and downregulated-ALKBH5 expressions (Fig. 1 H), in which ones also had the highest global m^6^A levels as compared with those with merely upregulated-METTL3 or downregulated-ALKBH5 (Fig. 1I). In established cell lines, all the tested LUAD cell lines acquired higher global m^6^A levels in comparison to either BEAS-2B cells, a lung epithelial cell line or 16HBE, a bronchial epithelial cell line (Supplementary Fig. 1 H-I); this might also because of upregulated-METTL3 and downregulated-ALKBH5 expressions (Supplementary Fig. 1 J). Among the LUAD cell lines, H1975 cells had the highest global m^6^A level while H1299 cells had the lowest m^6^A level (Supplementary Fig. 1 H). METTL3 was knocked out in H1975 cells while was ectopically expressed in H1299 cells. By contrast, ALKBH5 was overexpressed in H1975 cells while was knocked out in H1299 cells. Global m^6^A levels were reduced to the lowest level in H1975 cells with simultaneous METTL3 silencing and ALKBH5 overexpression compared with levels in cells with modulation of only one protein (Fig. 1 J). The opposite outcome was observed once upon METTL3 and ALKBH5 were simultaneously overexpressed and knocked out in H1299 cells (Fig. 1 K). However, the global m^6^A levels were unlikely regulated by METTL3 and ALKBH5 in BEAS-2B cells, suggesting that the regulation of m^6^A levels in lung epithelial cells might be different from that in LUAD cells. We also found that the stimulation of global m^6^A levels from overexpression of METTL14 and WTAP and knockout of FTO were not as obvious as those by overexpression of METTL3 and knockout of ALKBH5 in H1299 cells (Fig. 1 K and Supplementary Fig. 1 K-L). Together, these results strongly indicated that m^6^A levels in LUAD are regulated by METTL3 and ALKBH5.

We next examined the clinical outcome of LUAD patients with varied METTL3 and ALKBH5 expressions in cohort #2, which included patients that were followed up for 120 months following curative surgery. Patients with both upregulated-METTL3 and downrergulated-ALKBH5 still accounted for the largest proportion of the LUAD patient group (45.2%, 84/186, Fig. 1 L). These LUAD patients showed a shorter survival compared with patients with only upregulated-METTL3 or downregulated-ALKBH5 (Fig. 1 M).

### m^6^A-dependent pro-glycolytic outcomes are synergized by upregulation of METTL3 and downregulation of ALKBH5

**Fig. 2:**
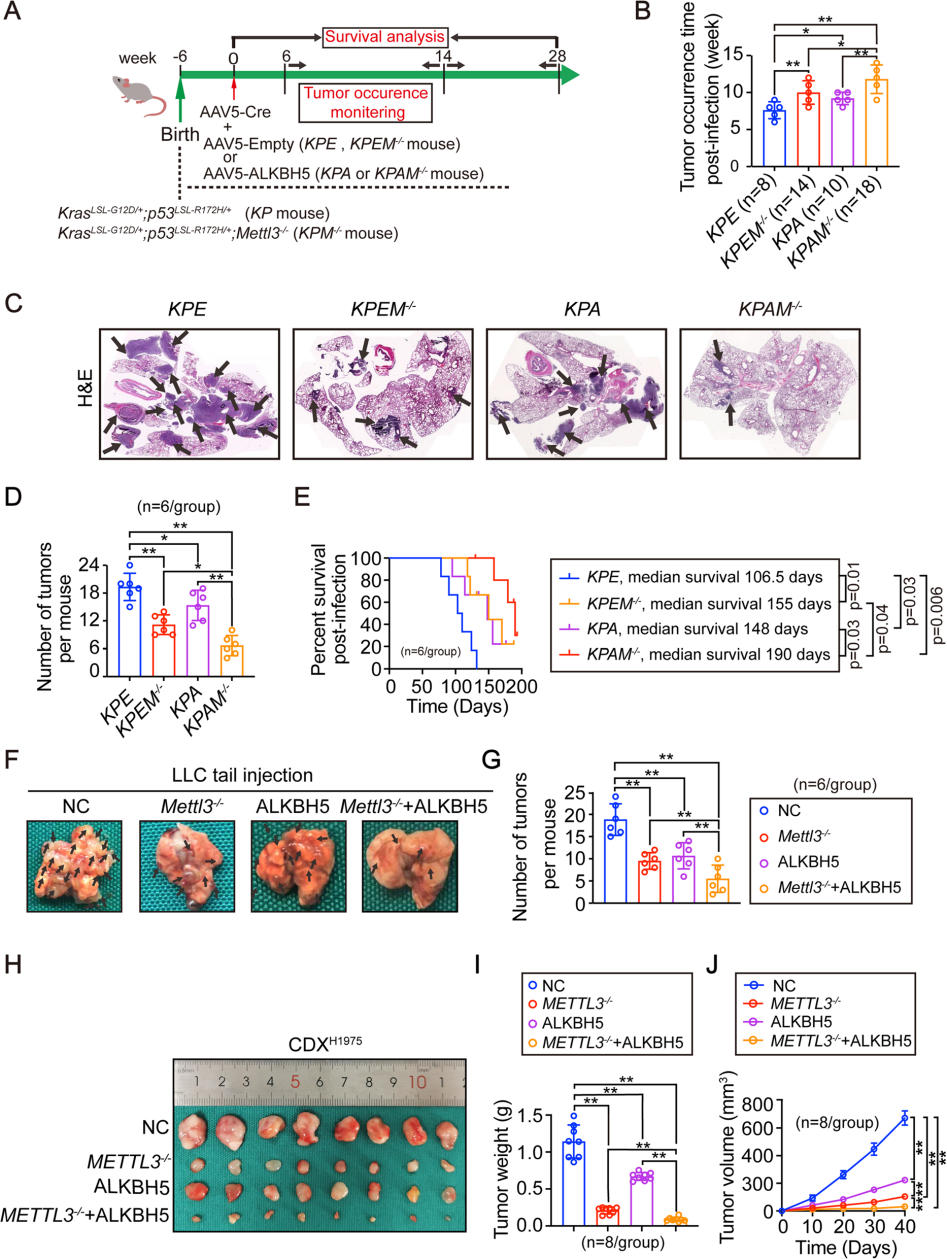
The roles of METTL3 and ALKBH5 in LUAD tumorigenesis.(**A**) Schematic presentation of the construction of *KP*-based mice models and the principle timeframe for the experiments. (**B**) Tumor occurrence time in different *KP*-based mice models, as indicated. Mice were monitored from the 6^th^ to 14^th^ weeks post infection. (**C**) Representative images of H&E staining for lung bearing tumors in indicated *KP*-based mice models. Black arrow indicates tumor foci. (**D**-**E**) Number of tumors (**D**) and overall survival (**E**) in indicated *KP*-based mice models (n=6/group). (**F**-**G**) Representative images of lungs bearing tumors (**F**) and numbers of tumors in lung (**G**) from mice following tail injection of LLC cells with distinct modulations, as indicated. n=6/group, black arrow indicates tumor. (**H**-**J**) Images of xenografts that generated by H1975 cells under different modulations (**H**). The tumor weights (**I**) and volume (**J**) were also examined (n=8/group). Statistical analysis was performed using one-way ANOVA (B, **D**, **G**, **I**), log-rank test (**E**) and two-way ANOVA (**J**). Data are presented as means ± SEMs from indicated samples. **p < 0.01 and *p<0.05 indicates statistical significance

The above findings showed that the combination of upregulated-METTL3 and downregulated-ALKBH5 was associated with highest global m^6^A levels in LUAD (Fig. 1). We next explored whether this combination has pro-tumorigenic functions in *KP* mice, which are useful models to study LUAD *in vivo* [25, 26]. *Mettl3* was further knocked out in *KP* mice to establish *KPM*^*−/−*^ mice. *KP* and *KPM*^*−/−*^ mice were then intranasally infected with AAV5-Cre to initiate LUAD and co-infected with empty or AAV5 expressing ALKBH5 to generate *KPE*, *KPA*, *KPEM*^*−/−*^, and *KPAM*^*−/−*^ mice, respectively (Fig. 2 A and Supplementary Fig. 2 A). Tumor growth was monitored for 9 weeks after infection. We found that the occurrence of intra-pulmonary tumors in *KPAM*^*−/−*^ mice was markedly later than that in *KPE*, *KPEM*^*−/−*^ and *KPA* mice (Fig. 2B). The tumor burden and numbers of tumor foci in lung were also much reduced following either *Mettl3* knockout (*KPE* vs. *KPEM*^*−/−*^) or ALKBH5 overexpression (*KPE* vs. *KPA*), and greater effects were observed in the group with both *Mettl3* knockout and ALKBH5 upregulation compared with that in mice modulated for either protein alone (*KPEM*^*−/−*^ and *KPA* vs. *KPAM*^*−/−*^, Fig. 2 C-D). Moreover, mice with both *Mettl3* knockout or ALKBH5 upregulation showed the longest survival time compared with that of the other experimental groups (Fig. 2E). These results indicated that the combination of upregulated METTL3 and downregulated ALKBH5 plays powerful pro-tumorigenic effects in LUAD.

*KPAM*^*−/−*^ mice are spontaneous LUAD models, which can reflect the opposite capacities of Mettl3 and ALKBH5 to transform lung epithelium to LUAD *in vivo*. To evaluate the roles of Mettl3 and ALKBH5 in affecting the malignancy of established lung cancer cells *in vivo* Mettl3 and ALKBH5 were pre-knocked out and pre-overexpressed separately or in combination in LLC cells, a murine lung cancer cell line, before tail injection. Synergized suppression of intra-pulmonary tumor formation was observed upon knocking out *Mettl3* and overexpressing ALKBH5 simultaneously (Fig. 2 F-G and Supplementary Fig. 1B), indicating that Mettl3 and ALKBH5 are critical for maintaining transformative phenotypes in murine lung cancer cells.

After elucidating the critical roles of Mettl3 and ALKBH5 in mice and murine lung cancer cells, we next evaluated their functions in human LUAD cells. As shown in Supplementary Fig. 2 C-D, either knocking METTL3 out or overexpressing ALKBH5 led to a suppression of cell viability in H1975 cells. While increased cell viability was observed in H1299 cells with either METTL3 overexpression or ALKBH5 knockout (Supplementary Fig. 2E-F). Similar outcomes were observed in 3D culture conditions and CDX mouse models (Fig. 2 H-J and Supplementary Fig. 2G-H). Expectedly, co-manipulating METTL3 and ALKBH5 synergized those effects (Fig. 2H-J and Supplementary Fig. 2 C-H). These data provided additional experimental evidences showing that the roles of METTL3 and ALKBH5 are conserved among species.

### ENO1 is associated with m^6^A levels to boost LUAD progression

**Fig. 3:**
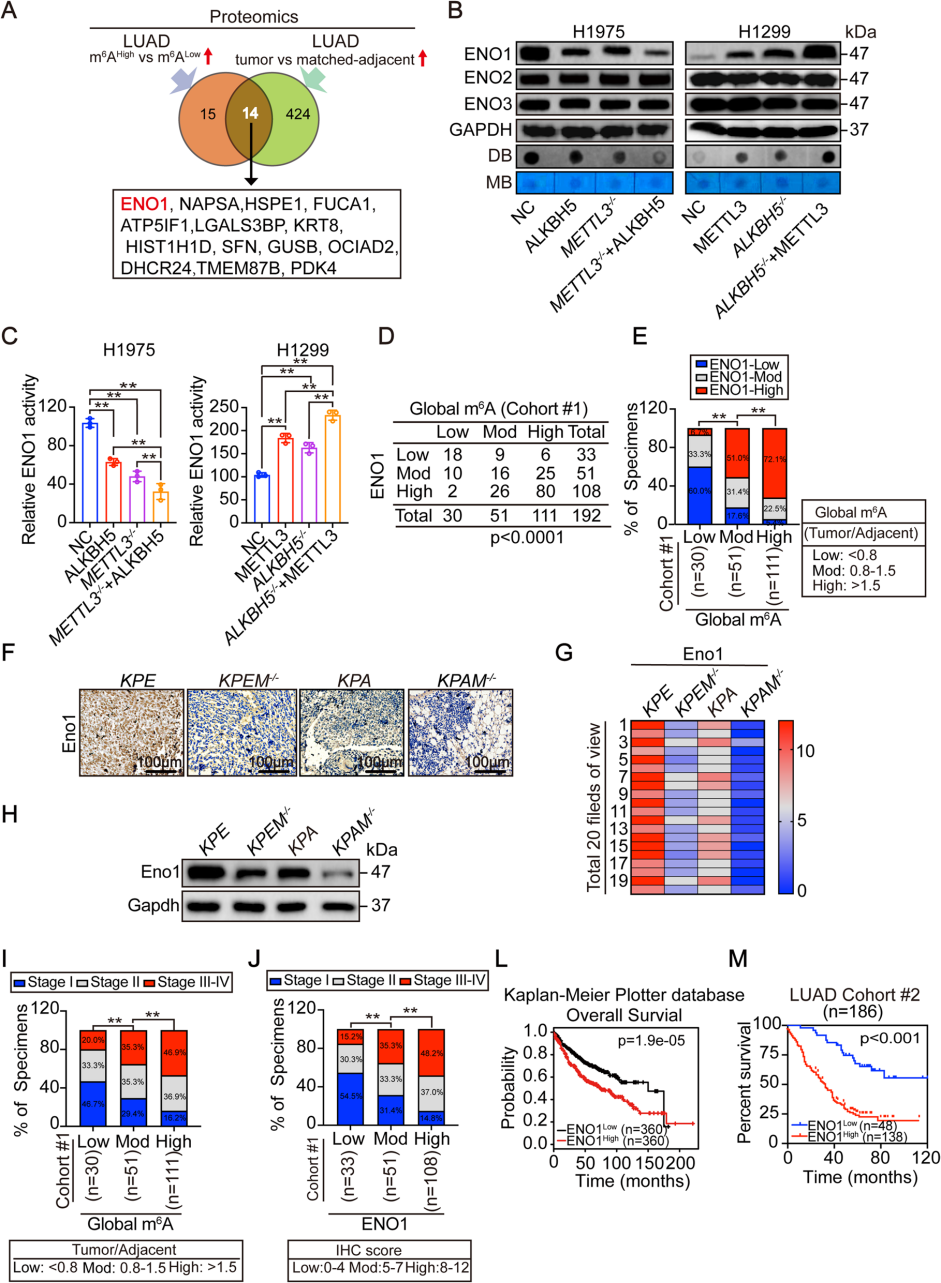
Association between m^6^A and ENO1 and the link with clinical outcome. (**A**) Venn diagram of proteomics showing candidates that were elevated in LUAD and upregulated by m^6^A. (**B**) ENO family expression and m^6^A levels in H1975 and H1299 cells with indicated treatment, as measured by IB and dot blot, respectively. (**C**) ENO1 activity in H1975 and H1299 cells with different treatments, as indicated. (**D**) Correlation between global m^6^A and ENO1 in LUAD. (**E**) The percentage of LUAD expressing different levels of ENO1 in those with different tumor/adjacent global m^6^A ratios. (F-H) IHC (**F**), heatmap (**G**) and IB (**H**) showing Eno1 expression in spontaneous LUAD from indicated *KP*-based mice. In panel G, the average levels of Eno1 in 20 fields of view are shown. Scale bar, 100 μm. (**I**-**J**) The percentage of patients at different stages in cohort #1 with different levels of global m^6^A (**I**) and ENO1 (**J**). (**L**-**M**) Overall survival of LUAD patients with high and low levels of ENO1, as analyzed from the data from Kaplan-Meier Plotter database (**L**) and our own (**M**) by log-rank test. Statistical analysis was performed using one-way ANOVA (**C**) and *Chi*-squared test (**E**, **I**, **J**). Data are presented as means ± SEMs from three independent experiments (**C**). **p < 0.01 indicates statistical significance

The combination of upregulated METTL3 with downregulated ALKBH5 elevates global m^6^A levels and stimulates tumorigenesis in LUAD (Figs. 1 and 2). We next explore the potential m^6^A effectors that are critical for LUAD. By proteomics, 29 proteins were identified to be upregulated in LUAD with higher m^6^A levels as compared to those with lower levels. Additionally, 438 proteins were elevated in tumors compared with matched adjacent tissues from LUAD patients. A total of 14 proteins overlapped between these two sample sets, and we ranked these 14 proteins using the average fold change and p value from the two independent proteomic experiments (Fig. 3 A and Supplementary Fig. 3 A). The top five ranked proteins, enolase1 (ENO1), napsin A aspartic peptidase (NAPSA), heat shock protein family E member 1 (HSPE1), alpha-L-fucosidase 1 (FUCA1) and ATP synthase inhibitory factor subunit 1 (ATP5IF1), were further analyzed in established lung epithelial BEAS-2B cells and LUAD H1299 and H1975 cells. Similar to the proteomics (Fig. 3 A), these 5 proteins were all upregulated in H1299 and H1975 cells in comparison with BEAS-2B cells (Supplementary Fig. 3B). To investigate whether the five proteins are regulated by m^6^A, METTL3 and ALKBH5 were knocked out and overexpressed in H1975 and H1299 cells. The single manipulating of METTL3 or ALKBH5 expression had no influence on HSPE1, FUCA1 and ATP5IF1 protein expression, while the combined manipulation of METTL3 and ALKBH5 significantly altered the expression of all five proteins (Supplementary Fig. 3 C-D). ENO1 was the top ranked protein and showed most sensitivity to the alteration of METTL3 and ALKBH5 (Fig. 3 A and Supplementary Fig. 3 A-D). Therefore, ENO1 was selected for subsequently study.

**Fig. 4:**
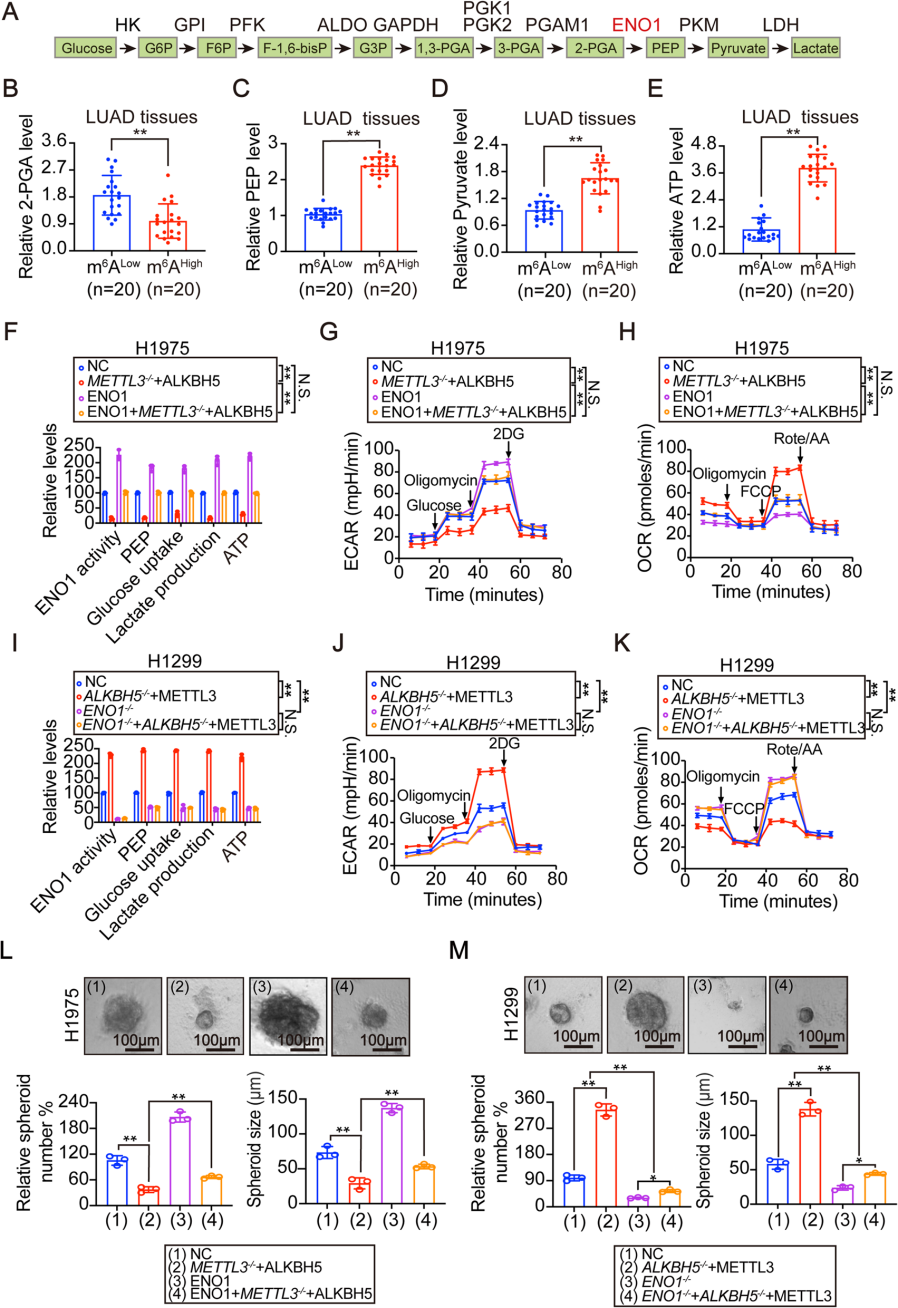
M^6^A-dependent regulation of glycolysis and 3D-spheroid formation via ENO1.Schematic representation of glycolysis processes. (**B**-**E**) The 2-PGA (**B**), PEP (**C**), Pyruvate (**D**) and ATP (**E**) levels in LUAD tissues with low or high global m^6^A levels. (**F**-**H**) ENO1 activity (**F**), PEP (**F**), glucose uptake (**F**), lactate production (**F**), ATP (**F**), ECAR (**G**) and OCR (**H**) in the presence or absence of METTL3 knockout and ALKBH5 overexpression, with or without ENO1 compensation in H1975 cells, as indicated. (**I**-**K**) ENO1 activity (**I**), PEP (**I**), glucose uptake (**I**), lactate production (**I**), ATP (**I**), ECAR (**J**) and OCR (**K**) in control and *ENO1*^-/-^ H1299 cells with or without ALKBH5 knockout and METTL3 overexpression. (**L**) 3D-spheroid formation that generated from H1975 cells with or without METTL3 knockout and ALKBH5 overexpression, in the presence or absence of compensation for ENO1. Scale bar, 100 μm. (**M**) 3D-spheroid formation that generated from control and *ENO1*^-/-^H1299 cells with or without ALKBH5 knockout and METTL3 overexpression. Scale bar, 100 μm. Statistical analysis was performed using t test (**B**-**E**), one-way ANOVA (**F**, **I**, **L**, **M**) and two-way ANOVA (**G**, **H**, **J**, **K**). Data are presented as means ± SEMs from indicated samples or three independent experiments. **p < 0.01 indicates statistical significance and N.S. indicates no significance

We next investigated whether ENO1 is linked with m^6^A. Among the three ENO family members, i.e. ENO1, ENO2 and ENO3, only ENO1 expression was regulated by METTL3 and ALKBH5 in H1975 and H1299 cells (Fig. 3B). Data from clinical LUAD specimens also demonstrated a definite m^6^A-ENO1 regulatory relationship, but not for ENO2 and ENO3 (Supplementary Fig. 3E). A significant correlation between global m^6^A levels and ENO1 protein expression was also revealed among LUAD cell lines (Supplementary Fig. 3 F). Therefore, the control of other two members in the m^6^A regulatory system was excluded. Besides expression, the activity of ENO1 could also be regulated by METTL3 and ALKBH5 (Fig. 3 C). Like global m^6^A (Fig. 1 J-K), stimulation to ENO1 was synergized by combined overexpressing METTL3 and knocking out ALKBH5 in H1299 cells (Fig. 4B-C). A tissue microarray assay (TMA) and data from Fig. 1 A revealed a significant global m^6^A levels-ENO1 correlation in LUAD (Fig. 3D-E and Supplementary Fig. 3 F G). We also observed a greater downregulation of Eno1 in *KPAM*^*−/−*^ compared with levels in the other mouse groups (Fig. 3 F-H). Furthermore, the levels of global m^6^A and ENO1 were both associated with tumor stage progression in LUAD (Fig. 3I-J). In Fig. 1M, we’ve demonstrated that a higher global m^6^A level resulted from upregulated-METTL3 and downregulated-ALKBH5 was indicative of a poorer overall survival. Here, the data not only from Kaplan-Meier Plotter public database [27], but also from ours, i.e. cohort #2, all supported that ENO1 is equally important to determine overall survival (Fig. 3 L-M). Together, these data indicated that ENO1 is associated with m^6^A levels and their functions to clinical outcome are closely linked with each other in LUAD.

As known, the function of ENO1 is determined by its subcellular localization [28]. Cytoplasmic localization of ENO1 was detected in 70.3% (135/192) of tested LUAD specimens and 29.7% (57/192) of LUAD specimens demonstrated a nuclear ENO1 subcellular localization (Supplementary Fig. 3H). Nuclear-ENO1 acts as a transcription factor to stimulate *c-MYC* transcription [29]. We found that alteration of m^6^A by METTL3 and ALKBH5 had no impact on *c-MYC* mRNA expression in H1975 and H1299 cells (Supplementary Fig. 3I). However, combined METTL3 overexpression and ALKBH5 knockout facilitated restriction of ENO1 in the cytoplasm (Supplementary Fig. 3 J). These results suggest that cytoplasmic ENO1 may be involved in m^6^A-boosted LUAD tumorigenesis.

### Increased m^6^A levels lead to increased glycolysis via ENO1

Previous studies showed that cytoplasmic ENO1 functions in modulating glycolysis [30, 31]. We therefore hypothesized that change in m^6^A levels may promote LUAD development through influencing glycolysis. ENO1 catalyzes the generation of PEP from 2-PGA (illustrated in Fig. 4 A and Ref. [19, 32]). If the role of m^6^A levels is ENO1-dependent, a high level of m^6^A could facilitate 2-PGA consumption and stimulate generation of PEP and its downstream metabolites. We thus compared metabolites between LUAD tissues with low and high global m^6^A levels. Except for a reduction of 2-PGA, the inductions of PEP and its downstream pyruvate were observed in LUAD specimens with a higher global m^6^A level (Fig. 4B-D). Release of ATP is not a specific event for glycolysis, however, we found that ATP release was elevated by m^6^A levels (Fig. 4E). In the analysis of the same LUAD specimens from Fig. 1I, an increased level of global m^6^A was found to elevate concentrations of PEP, pyruvate and ATP and reduce 2-PGA (Supplementary Fig. 4 A). Besides, except ENO1, the expressions of other enzymes in the glycolysis were unaffected by m^6^A levels in H1975 and H1299 cells (illustrated in Fig. 4 A and Supplementary Fig. 4B-C). These data hinted a role of m^6^A levels in stimulating glycolysis via ENO1  in LUAD.

We next examined whether the suppression of glycolysis caused by reducing m^6^A levels could be rescued by ENO1 in human LUAD cells. To this end, m^6^A levels were suppressed by METTL3 knockout and ALKBH5 overexpression before compensating with or without ENO1 in H1975 cells. The suppressed-global m^6^A levels were not rescued by ENO1 (Supplementary Fig. 4D-E), suggesting that ENO1 cannot feedback regulate m^6^A. However, the redcued ENO1 activity could be fully compensated in H1975 cells (Fig. 4 F). Besides PEP and ATP, glucose uptake and lactate production were also examined. The results demonstrated glycolysis inhibition following m^6^A suppression, and this was rescued by ENO1 (Fig. 4 F). Glycolysis is accompanied by an increase of ECAR and a decrease of OCR [33]. Suppression of m^6^A levels led to a significant reduction of ECAR but an induction of OCR, which was rescued by ENO1 (Fig. 4G-H). These results suggested that a reduction of ENO1 is a prerequisite for the inactivation of glycolysis following suppression of m^6^A levels.

To further support the important role of ENO1 for m^6^A-stimulated glycolysis, we pre-knocked out ENO1 in H1299 cells examined glycolysis following overexpression of METTL3 and knocking out ALKBH5. Global m^6^A levels were still elevated even when ENO1 was knocked out (Supplementary Fig. 4F-G). However, while ENO1 activity was reduced to an almost undetectable level following ENO1 knockout, this was not rescued by overexpressing MELLT3 and knocking ALKBH5 out at all (Fig. 4I), suggesting that m^6^A levels are ineffective in modulating ENO1 in the absence of this target itself. Furthermore, loss of ENO1 eliminated the capacity of m^6^A to stimulate glycolysis (Fig. 4I-K). Thus, these data once again elucidated that ENO1 is the genuine target of m^6^A to boost glycolysis in LUAD cells.

Then, we investigated whether ENO1-dependent m^6^A stimulation of glycolysis is powerful enough to influence transformative phenotypes in 3D-cultured LUAD cells. We found that cell proliferation and invasion abilities were positively modulated by m^6^A levels in H1975 and H1299 cells in an ENO1-dependent manner (Fig. 4 L-M, Supplementary Fig. 4 H-K). Thus, we speculated that m^6^A levels induce tumorigenesis at least in part through its control of ENO1-dependent glycolysis in LUAD cells.

### M^6^A methylation stimulates translation of ENO1 and glycolysis via 359 A in LUAD cells

**Fig. 5:**
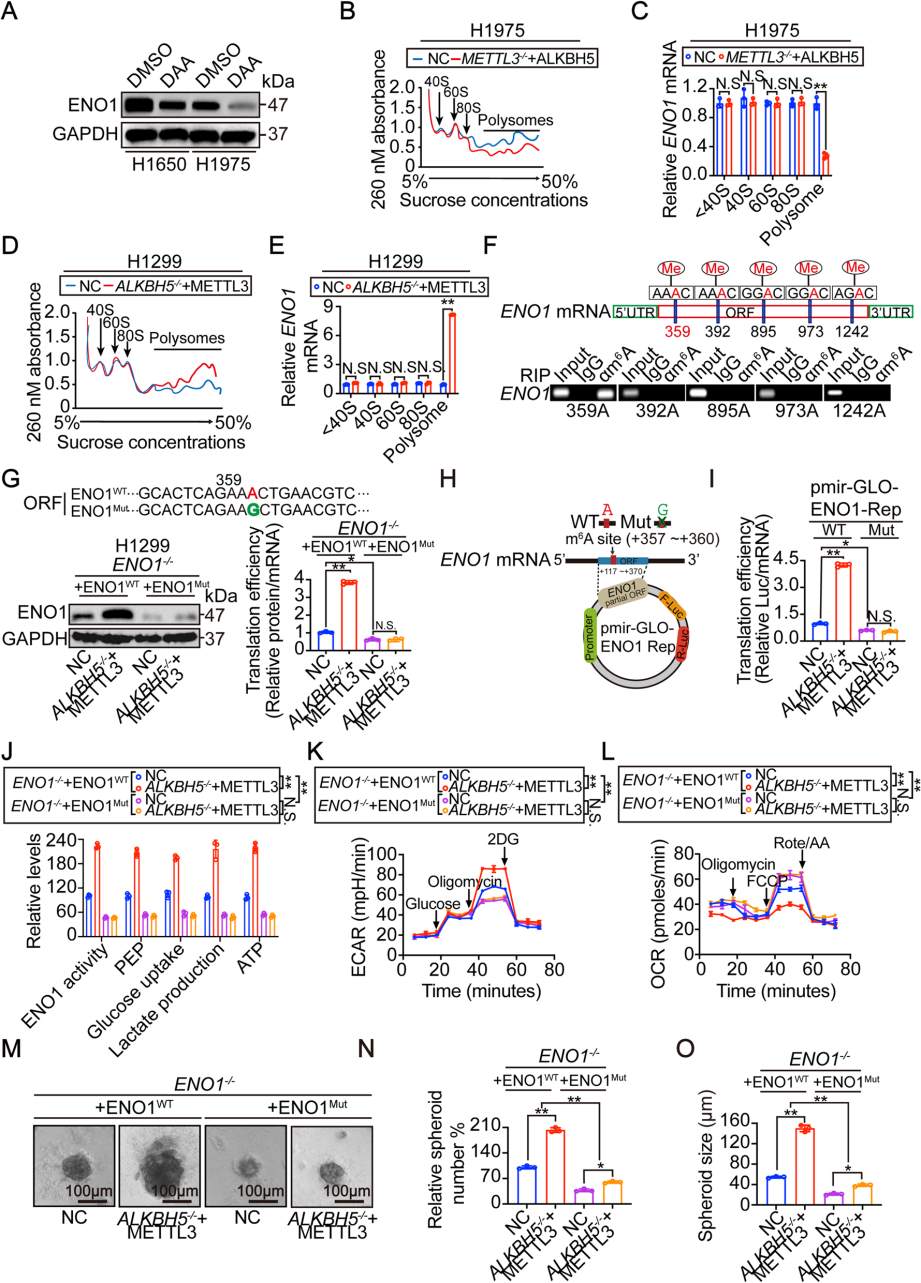
M^6^A methylation of *ENO1* mRNA was critical for its translation and glycolysis.(**A**) Representative IB images of ENO1 in H1650 and H1975 cells treated with DMSO or DAA (100 μM, 24h). (**B**) Polysome profiling in H1975 cells with or without combined METTL3 knockout and ALKBH5 overexpression. (**C**) Ribosome-associated *ENO1* mRNA in H1975 cells with or without combined METTL3 knockout and ALKBH5 overexpression. (**D**) Polysome profiling in H1299 cells with or without combined ALKBH5 knockout and METTL3 overexpression. (**E**) Ribosome-associated *ENO1* mRNA in H1299 cells with or without combined ALKBH5 knockout and METTL3 overexpression. (**F**) Prediction and verification of potential m^6^A sites within *ENO1* mRNA, as predicted by SRAMP online software and verified by RIP experiments using anti-m^6^A antibodies. (**G**) ENO1 protein expression and translation efficiency in *ENO1*^-/-^ H1299 cells that reconstituted with WT or Mut ENO1 (359A to 359G), with or without combined ALKBH5 knockout and METTL3 overexpression. (**H**) Schematic presentation of the construction of the pmir-GLO-ENO1 reporter containing *ENO1* partialORF region with or without 359A mutation. (**I**) Translation efficiency of *ENO1-LUC* fusion mRNA, as calculated by the ratios between luciferase activities and mRNA levels in H1299 cells with or without combined ALKBH5 knockout and METTL3 overexpression. (**J**-**L**) ENO1 activity (**J**), PEP (**J**), glucose uptake (**J**), lactate production (**J**), ATP (**J**), ECAR (**K**) and OCR (**L**) in *ENO1*^-/-^ H1299 cells reconstituted with WT or Mut ENO1 (359A to 359G), with or without combined ALKBH5 knockout and METTL3 overexpression. (**M**-**O**) Representative images (**M**), number (**N**) and size (**O**) of 3D-spheroids that generated by *ENO1*^-/-^ H1299 cells reconstituted with WT or Mut ENO1 (359A to 359G), with or without combined ALKBH5 knockout and METTL3 overexpression. Scale bar, 100 μm. Statistical analysis was performed using t test (**C**, **E**), one-way ANOVA (**G**, **I**, **J**, **N**, **O**) and two-way ANOVA (**K**, **L**). Data are presented as means ± SEMs from three independent experiments. **p < 0.01, *p<0.05 indicates statistical significance and N.S. indicates no significance

Our results indicate that ENO1 is regulated by m^6^A (Fig. 3). We further explored the underlying mechanism. Treatment of DAA, a pan-methylation inhibitor, resulted in a significant reduction of ENO1 protein but not mRNA in LUAD H1650 and H1975 cells (Fig. 5A and Supplementary Fig. 5A). At genetic levels, alteration of METTL3 and ALKBH5 did not influence *ENO1* mRNA expression in H1975 and H1299 cells (Supplementary Fig. 5B-C). An influence of METTL3 and ALKBH5 on the decay of *ENO1* mRNA was also excluded (Supplementary Fig. 5D-G). CHX chase experiments showed that the half-lives of ENO1 protein were comparable between H1975 and H1299 cells (Supplementary Fig. 5H), although the global m^6^A levels were varied (Supplementary Fig. 1H-I). Experiments in H1975 and H1299 cells with alteration of METTL3 and ALKBH5 also demonstrated that m^6^A-dependent regulation of ENO1 protein did not occur by the regulation of protein half-life (Supplementary Fig. 5I). We next examined whether m^6^A-dependent regulation of ENO1 protein involves translation. Polysome profiling analysis demonstrated that only ploysomes, but not 80S monosome, 40S and 60S ribosome subunits were reduced following combined METTL3 knockout and ALKBH5 overexpression in H1975 cells (Fig. 5B). By assessing the association of *ENO1* mRNA with the ribosome, we observed that only the association of *ENO1* mRNA to the polysome was reduced by suppression of m^6^A levels in H1975 cells (Fig. 5C). By contrast, increased polysome concentration and *ENO1* mRNA association with polysome were found following lifting m^6^A by combined ALKBH5 knockout and METTL3 overexpression in H1299 cells (Fig. 5D-E). Moreover, the translation efficiency of ENO1 was increased by m^6^A in a dose-dependent manner in H1299 cells (Supplementary Fig. 5J). These data suggested that ENO1 protein is upregulated by m^6^A through increased translation efficiency.

Previous studies showed that the translation of target mRNAs is enhanced following m^6^A methylation of the ORF region [34]. Analysis using the SRAMP online software predicted, five potential m^6^A sites, i.e. 359A, 392A, 895A, 973A and 1242A, within the ORF of *ENO1* mRNA were predicted (Fig. 5F). RIP experiments using anti-m^6^A antibodies demonstrated that only the region around the 359A was positioned to be m^6^A methylated (Fig. 5F and Supplementary Fig. 5K). Moreover, enrichments of the region around the 359A site were changeable and the degrees were positively associated with the levels of m^6^A in H1975 and H1299 cells, these results were not observed in an unrelated control region (Supplementary Fig. 5L-M). To precisely elucidate the importance of 359A in the m^6^A-dependent regulation of ENO1 translation, we replaced the adenosine (A) with a guanosine (G), and reconstituted *ENO1*^-/-^ H1299 cells with this mutant other than the wild type (*WT*) ENO1. We found that once upon the 359A was replaced, the basal level of ENO1 protein and translation efficiency were significantly reduced, and notably, and the m^6^A-dependent induction of translation was totally blocked (Fig. 5G). Similar findings were observed in examining the translation efficiency of an exogenous *ENO1-LUC* fusion mRNA by testing luciferase activity and the mRNA level using a pmir-GLO-based luciferase reporter, which contains *WT* or mutant partial *ENO1* ORF cloned upstream the luciferase-coding region (Fig. 5H-I). Functional experiments showed that the 359A is also critical for sustaining ENO1 activity and m^6^A-dependent stimulation of glycolysis and 3D-spheroid formation (Fig. 5J-O). These results demonstrated that the 359A is essential for the m^6^A modification to stimulate ENO1 translation and function.

### The m^6^A reader YTHDF1 is essential for executing m^6^A-dependent stimulation of ENO1 translation and function

**Fig. 6:**
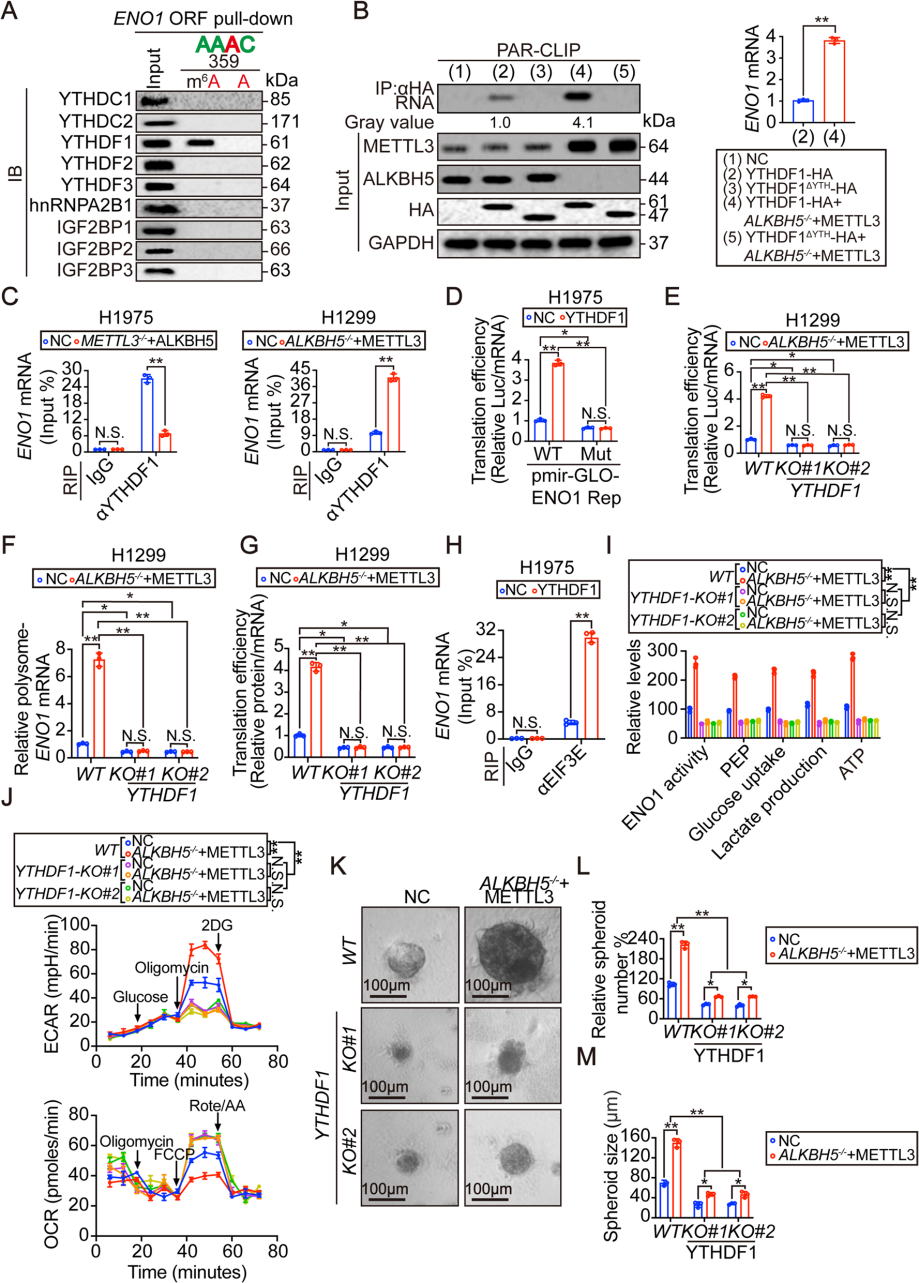
M^6^A reader YTHDF1 was essential for m^6^A to stimulate ENO1 translation and function.(**A**) Association of m^6^A readers, as indicated, with *ENO1* partial ORF region with or without artificially m^6^A-methylated 359A, as measured by IB following RNA pull-down experiment. (**B**) YTHDF1 interaction with *ENO1* mRNA, as measured by PAR-CLIP experiment using anti-HA antibodies in H1299 cells expressing HA-tagged YTHDF1 with or without YTH-domain, and treated with or without combined ALKBH5 knockout and METTL3 overexpression. RNA labeled with biotin was visualized by the chemiluminescent nucleic acid detection module. *ENO1* mRNA levels in the pulled down products were verified by qPCR. (**C**) Association between YTHDF1 and *ENO1* mRNA in H1975 cells with or without combined METTL3 knockout and ALKBH5 overexpression, and in H1299 cells with or without combined ALKBH5 knockout and METTL3 overexpression, as measured by RIP experiments using anti-YTHDF1 and IgG antibodies. (**D**) Translation efficiency of *ENO1-LUC* fusion mRNA in H1975 cells transfected with WT or Mut pmir-GLO-ENO1 reporter, and overexpressed with or without YTHDF1. (**E**) Translation efficiency of *ENO1-LUC* fusion mRNA in *WT* and *YTHDF1-KO* H1299 cells with or without combined ALKBH5 knockout and METTL3 overexpression. (**F**-G) Polysome-associated *ENO1* mRNA (**F**) and translation efficiency of endogenous *ENO1* mRNA (**G**) in *WT* and *YTHDF1-KO* H1299 cells with or without combined ALKBH5 knockout and METTL3 overexpression. (**H**) The recruitment of EIF3E at *ENO1* mRNA in H1975 cells with or without YTHDF1 overexpression, as measured by RIP using anti-EIF3E and control IgG antibodies. (**I**-**J**) ENO1 activity (**I**), PEP (**I**), glucose uptake (**I**), lactate production (**I**), ATP (**I**), ECAR (**J**) and OCR (**J**) in *WT* and *YTHDF1-KO* H1299 cells with or without combined ALKBH5 knockout and METTL3 overexpression. (K-M) Representative images (**K**), numbers (**L**) and size (**M**) of 3D-spheroids that generated by *WT* and *YTHDF1-KO* H1299 cells with or without combined ALKBH5 knockout and METTL3 overexpression. Scale bar, 100 μm. Statistical analysis was performed using t test (**B**, **C**, **H**), one-way ANOVA (**D**-**G**, **I**, **L**, **M**) and two-way ANOVA (**J**). Data are presented as means ± SEMs from three independent experiments. **p < 0.01, *p<0.05 indicates statistical significance and N.S. indicates no significance

M^6^A readers are terminal effectors for m^6^A methylation [35, 36]. We next investigated the m^6^A reader that is required for the translation of ENO1, by performing RNA pull-down assays using a partial *ENO1* ORF RNA with or without artificially modified m^6^A at 359 sites were performed. Several m^6^A readers including those belonging to the YTH family (YTHDC1, YTHDC2, YTHDF1, YTHDF2 and YTHDF3), IGF2BP family (IGF2BP1, IGF2BP2 and IGF2BP3) and hnRNPA2B1 were screened, and only YTHDF1 specifically bound with m^6^A-methylated *ENO1* mRNA (Fig. 6A). The YTH domain is essential for YTH family proteins to recognize m^6^A-methylated RNAs [37]. HA-tagged WT YTHDF1 and mutant YTHDF1 without the YTH-domain (YTHDF1^△YTH^) were expressed in H1299 cells before HA-tagged YTHDF1 was immunoprecipitated by anti-HA antibodies in PAR-CLIP experiments. The results showed that YTH-domain was required for YTHDF1 binding with RNA, and this interaction was increased by inducing m^6^A levels through ALKBH5 knockout and METTL3 overexpression in H1299 cells (Fig. 6B). In addition, the *ENO1* mRNA-YTHDF1 interactions was strengthened in response to induction of m^6^A in a dose-dependent manner (Fig. 6B and Supplementary Fig. 6A). RIP experiments further demonstrated that the *ENO1* mRNA-YTHDF1 interactions was positively regulated by m^6^A levels in H1975 and H1299 cells (Fig. 6C and Supplementary Fig. 6B). Together, these results indicate that YTHDF1 prefers binding with* ENO1* mRNA following m^6^A methylation.

Reasoning that m^6^A stimulates ENO1 translation (Fig. 5G-I), we wondered whether this is via YTHDF1. Using the luciferase reporter described in Fig. 5H, we found that the translation of ENO1 was induced by YTHDF1; however, it was blocked upon replacement of 359A with a G (Fig. 6D). Compared to the *WT* H1299 cells, knocking out YTHDF1 significantly reduced ENO1 protein expression and diminished the induction of ENO1 translation by lifting global m^6^A (Fig. 6E and Supplementary Fig. 6C), suggesting that m^6^A stimulated-ENO1 translation requires YTHDF1. We further evaluated endogenous *ENO1* mRNA, and found that the m^6^A-dependent inductions of *ENO1* mRNA-polysome association and ENO1 translation were all blocked following knocking out YTHDF1 (Fig. 6F-G). Sequential recruitment of a series of translation initiation factors, for example, eukaryotic translation initiation factor 3 (EIF3E), triggers translation of a certain target mRNA [38, 39]. We found that overexpressing YTHDF1 facilitated EIF3E recruitment to the *ENO1* mRNA (Fig. 6H). These data suggested that YTHDF1 is required for m^6^A-dependent stimulation of ENO1 translation..

We then functionally examined whether YTHDF1 is linked with glycolysis and the transformative phenotypes of LUAD cells. Increasing global m^6^A levels failed to induce ENO1 activity in H1299 cells with YTHDF1 knocked out (Fig. 6I). Similar findings were observed for the concentrations of PEP and ATP concentrations, glucose uptake, lactate production, ECAR and OCR (Fig. 6I-J), demonstrating that YTHDF1-associated stimulation of ENO1 activity is essential for m^6^A-induced glycolysis. YTHDF1 was also required for the m^6^A-dependent generation and growth of 3D-spheroids (Fig. 6K-M). Together, these results indicate that YTHDF1 is important for m^6^A-induced glycolysis and tumorigenesis in LUAD cells.

### Clinical and translational significance of the current study

**Fig. 7:**
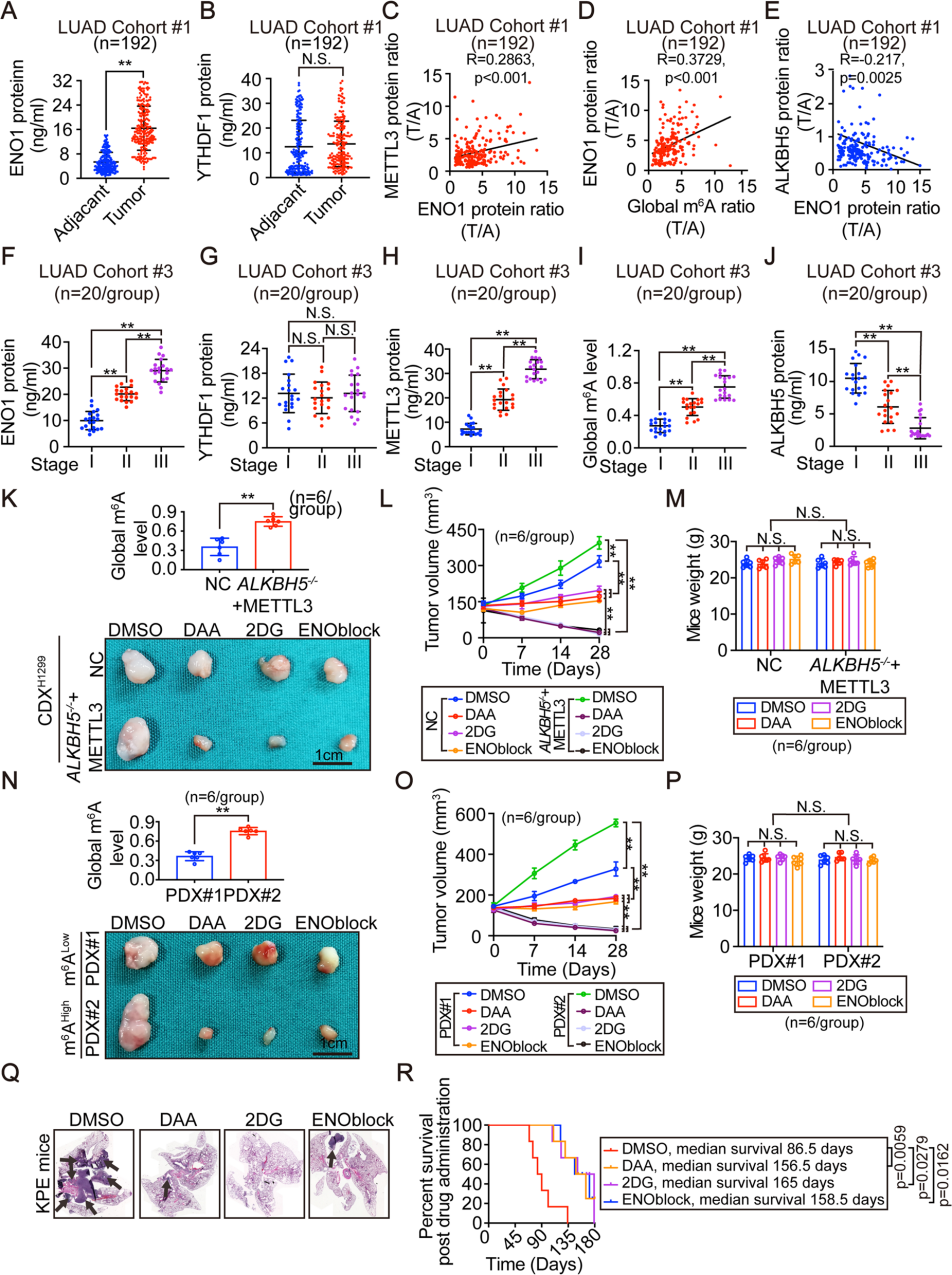
Clinical and translation significance of the study.(**A**-**B**) ENO1 and YTHDF1 protein expression in adjacent and matched-tumor tissues from LUAD patients of cohort #1 (n=192). (**C**-**E**) Correlations between METTL3 and ENO1 (**C**), ENO1 and global m^6^A (**D**), and between ALKBH5 and ENO1 (**E**) in cohort #1. The protein and m^6^A levels were calculated as the ratios between that from tumor and matched-adjacent tissues. (**F**-**J**) ENO1 (**F**), YTHDF1 (**G**), METTL3 (**H**), global m^6^A level (**I**) and ALKBH5 (**J**) in LUAD with indicated tumor stages from cohort #3 (n=20/group). (**K**-**M**) CDX that generated by H1299 cells with or without combined ALKBH5 knockout and METTL3 overexpression followed by administrating mice with DMSO, DAA (50mg/kg), 2DG (1000mg/kg) or ENOblock (20mg/kg). The global m^6^A levels (**K**), representative images of xenografts (**K**), tumor volume (**L**) and mice weights (**M**) were graphed and shown (n=6/group). Scale bar, 1cm. (**N**-**P**) PDX mice models with high and low global m^6^A levels were administrated with DMSO, DAA (50mg/kg), 2DG (1000mg/kg) or ENOblock (20mg/kg). The global m^6^A levels (**N**), representative images of xenografts (**N**), tumor volume (**O**) and mice weight (**P**) were graphed and shown. (n=6/group). Scale bar, 1cm. (**Q**-**R**) Representative H&E images of spontaneous LUAD in *KPE* mice following being infected with AVV5 expressing Cre and administrated with DMSO, DAA (25mg/kg), 2-DG (500mg/kg) or ENOblock (10mg/kg), as indicated (**Q**). The overall survival curves for KPE mice with established LUAD following drug administration are also shown in panel P R (n=6/group). Statistical analysis was performed using t test (**A**, **B**, **K**, **N**), spearman rank-correlation analysis (**C**-**E**), one-way ANOVA (**F**-**J**, **M**, **P**), two-way ANOVA (**L**, **O**) and log-rank tests (**R**). Data are presented as means ± SEMs from indicated samples. **p < 0.01 indicates statistical significance and N.S. indicates no significance

Our results demonstrated that the relationship between ENO1 and m^6^A plays critical roles to stimulate glycolysis and is pro-tumorigenic in established LUAD cells (Figs. 3, 4, 5 and 6). We next explored whether this mechanism occurs in human LUAD. We found that ENO1 was upregulated in LUAD specimens in cohort #1 (n=192) (Fig. 7A). However, the levels of YTHDF1 were similar in LUAD and matched adjacent tissues (Fig. 7B), suggesting that YTHDF1 regulation of ENO1 is merely followed by the alteration of global m^6^A levels. Combined with the data in Fig. 1A-D, further experiments showed that ENO1 positively correlated with METTL3 and global m^6^A levels and negatively correlated with ALKBH5 in LUAD (Fig. 7C-E), suggesting that an upregulated-ENO1 expression is indeed a result from elevated global m^6^A levels that is coordinately modulated by the upregulation of METTL3 and downregulation of ALKBH5. In cohort #3, which contains randomly recruited LUAD patients with accurate tumor progression information, we found that except YTHDF1, ENO1, METTL3 and global m^6^A levels were positively while ALKBH5 was negatively associated with tumor stages (Fig. 7F-J), further demonstrating the important roles of m^6^A and ENO1 in tumor progression.

Because activation of ENO1-dependent glycolysis is one of terminal effects from inducing global m^6^A levels (Figs. 4, 5 and 6), we wondered whether LUADs with higher global m^6^A levels are more sensitive to the inhibition to global m^6^A, glycolysis and ENO1. To address these questions, we constructed CDX mouse models generated from H1299 cells with or without combined knocking out of ALKBH5 and METTL3 overexpression. As expected, CDX models with varied global m^6^A levels were obtained, and the higher m^6^A levels in tumors were associated with faster tumor growth *in vivo* (Fig. 7K-L). We also treated mice with DAA (a pan-methylation inhibitor), 2DG (a glycolysis inhibitor) and ENOblock (a pan-ENO inhibitor), respectively, and found that tumor growth was suppressed more significantly and even regressed in tumors with higher m^6^A levels (Fig. 7K-L and Supplementary Fig. 7A). Notably, the mice bodyweights were not affected by the treatment (Fig. 7M). We also performed experiments in PDX mouse models, which more accurately reflect the real tumor situation of patients, were also used. We found that DAA, 2DG and ENOblock showed more significant inhibition against the tumor of PDX#2, which demonstrated a higher global m^6^A level than that of PDX#1 (Fig. 7 N-O and Supplementary Fig. 7B). The bodyweights of PDX-bearing mice were not affected by treatment (Fig. 7P). Furthermore, spontaneous LUAD in *KPE* mice was inhibited by DAA, 2DG and ENOblock (Fig. 7Q), and these mice showed improved survival (Fig. 7R). Together, these findings indicate that inhibition of m^6^A, glycolysis and ENO is helpful to treat LUAD, especially tumors with higher global m^6^A levels, with minimum toxicity.

## Discussion

**Fig. 8:**
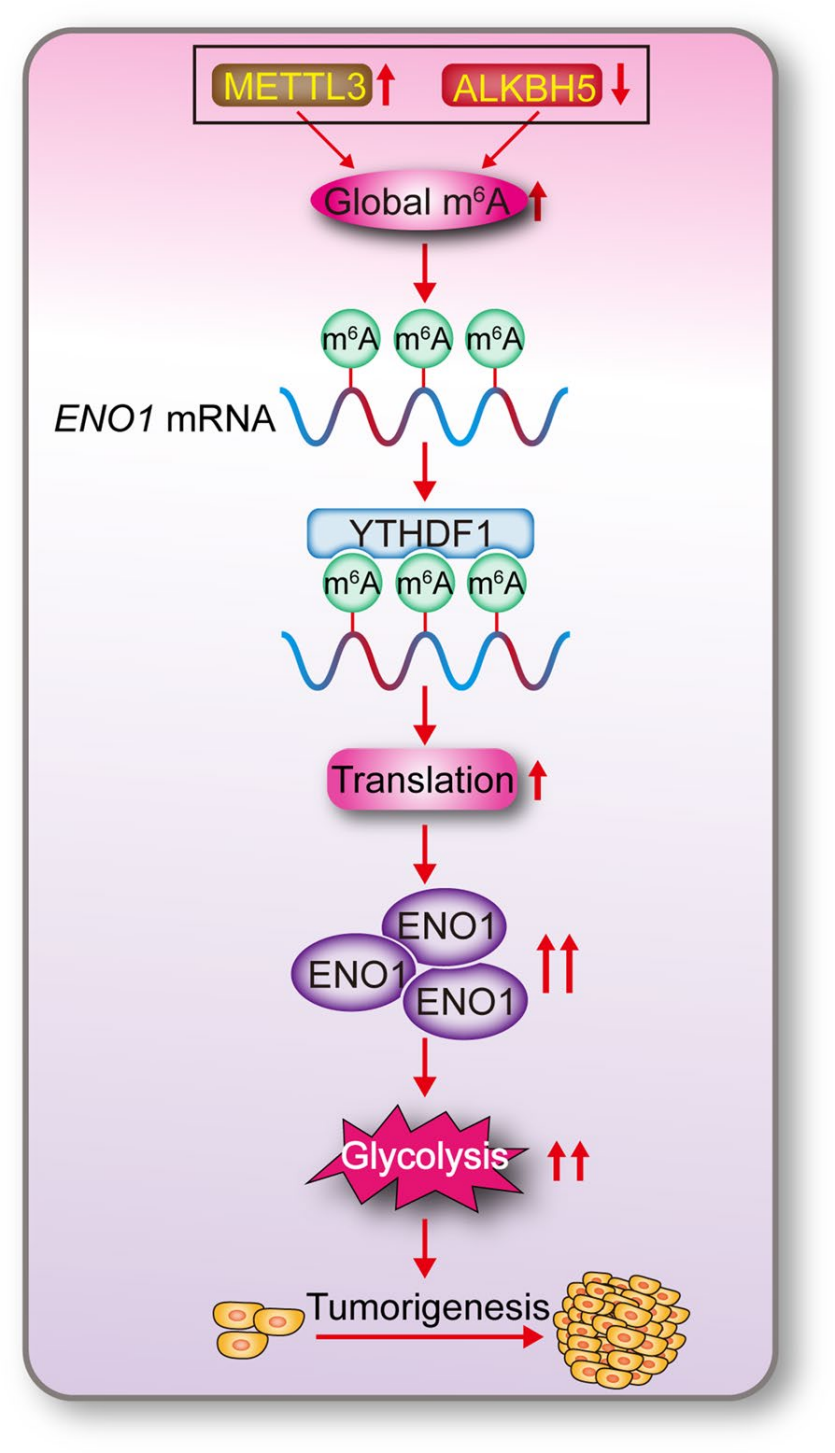
Schematic presentation of the study.Briefly, elevated-global m^6^A levels that determined by upregulated-METTL3 and downregulated-ALKBH5 facilitate m^6^A methylation of mRNA, such as *ENO1* in LUAD. M^6^A reader YTHDF1 is prone to interact with m^6^A-methylated *ENO1* mRNA, by which leads to a stimulation of ENO1 translation. Increased ENO1 in turn causes reinforcement in glycolysis and tumor growth in LUAD

As an epigenetic regulator, m^6^A modifications, mediated by the WER system, are abundant in RNA [40]. Previous studies have linked m^6^A modifications to the development of lung cancer [41]. Several reports showed that m^6^A writers act as oncogenic-proteins to elevate global m^6^A levels [42, 43], while m^6^A erasers decrease global m^6^A levels and act as tumor suppressors [44]. Compared to the writers and erasers, m^6^A readers are terminal effectors of the m^6^A modifications. Previous studies showed that the m^6^A readers YTHDC2 and YTHDF2, are critical for LUAD tumorigenesis [24, 45]. Increasing reports have demonstrated the importance of m^6^A modification and its regulators in lung tumorigenesis. For example, METTL3 is upregulated in lung cancer and required for tumor growth, invasion, survival and progression [46, 47], while ALKBH5 has the opposite effects [44]. However, to the best of our knowledge, existing studies of the WER system in LUAD are relatively scattered, and no study has comprehensively delineated how the WER system is orchestrated and functions in the tumorigenesis of LUAD. In the present study, our findings establish a model underlying the coordination of m^6^A modification by the WER to stimulate LUAD tumorigenesis (Fig. 8). We demonstrated that the elevated global m^6^A levels in LUAD are the results of the combined upregulation of the writer METTL3 and the downregulation of the eraser ALKBH5 in a large proportion of the LUAD specimens. The roles of the reader YTHDF1 to stimulate translation of ENO1 and subsequent glycolysis are essential for m^6^A modifications to boost LUAD tumorigenesis.

In this study, we found that *ENO1* mRNA is m^6^A methylated in LUAD, and this is also essential for the stimulation of ENO1 translation. Cytoplasmically localized ENO1 functions as a metabolic enzyme that participates in glycolysis [48]. We found that ENO1 was localized in the cytoplasm of most LUAD specimens, which is consistent with the findings from other studies demonstrating that ENO1 majorly acts to stimulate glycolysis in the cytoplasm [48]. A truncated version of ENO1 with a molecular weight of 37 kDa is also be translated from *ENO1* mRNA [49]. This protein tends to be localized in the nucleus and binds the *c-MYC* promoter, functioning as a tumor suppressor [50]. Whether ENO1 tends to be translated from full length of *ENO1* mRNA in LUAD has not yet been verified and needs to be investigated in the future. However, we found that the transcription of *c-MYC*, which is driven by nuclear ENO1, is largely not m^6^A-dependent, indicating that ENO1 may not act as a nuclear transcription factor in LUAD. ENO1 is upregulated in various cancers, and the mechanism depends on the cancer type. For example, ENO1 is upregulated via WW domain binding protein2 (WBP2) in glioma cancer cells [51]; however, the upregulation of ENO1 is ubiquitin-dependent in colorectal cancer cells [52]. Here, we show that the ENO1 is upregulated in LUAD cells through an m^6^A-dependent mechanism. Hence, our findings expend our understanding of the complicated regulatory mechanism of ENO1.

ENO1 is involved inmultiple pro-tumorigenic and pro-glycolytic processes, such as but not limited to tumor growth, metastasis and migration [53]. ENO1 functions via activating Wnt/β-catenin, AMPK/mTOR and PI3K/AKT signaling in lung tumorigenesis [54–56]. However, no study has linked ENO1 with m^6^A modification in LUAD. In other words, we are able to provide another proof supporting that the occurrence of LUAD is not a merely result from disorder of a single signaling cascade.

Active glycolysis is essential for tumor growth in both aerobic and hypoxic environments, with high rates of glucose uptake and lactate production regardless of the oxygen supply [57]. This metabolic reprogramming supports cancer cell growth by supplying resources to support the excessive energy demands of tumors [58]. A prior study demonstrated that m^6^A modifications of *c-MYC *mRNA promote glycolysis and tumor growth in LUAD [59]. Our study also establishes the close relationship between the METTL3-ALKBH5-YTHDF1 m^6^A axis and ENO1 as another important stimulator of m^6^A-dependent glycolysis. Increased glycolysis is a hallmark of tumor progression. Therefore, the role of m^6^A in glycolysis demonstrated here suggests an important mechanism in the regulation of LUAD tumorigenesis.

The destinies of mRNA transcripts with m^6^A modifications is determined by the diverse m^6^A readers [7]. For example, the m^6^A reader YTHDC2 accelerates the decay of target mRNAs [23, 24]. In contrast, those readers belonging to the IGF2BP family tend to prolong the half-lives of specific mRNAs after m^6^A methylation [8, 60]. Unlike other readers, YTHDF1 promotes ribosome loading and translation of m^6^A methylated mRNAs [61, 62]. Our findings showed that the translation of ENO1 is promoted by YTHDF1. Notably, YTHDF1 has been linked with NSCLC progression [63]. Therefore, the m^6^A-mediated LUAD progression identified in this study might occur in an YTHDF1-dependent manner.

Given the importance of m^6^A and ENO1-dependent glycolysis in the tumorigenesis and progression of LUAD, the inhibition of m^6^A, glycolysis and ENO1 may represent new therapeutic strategies for those LUAD patients with higher m^6^A levels. Emerging studies have revealed that methylation inhibitors, such as DAA or glycolysis inhibitors, such as 2DG are capable of suppressing transformative phenotypes *in vitro* [64, 65]. However, it is unknown how the effects of these inhibitors in LUAD. Our results from CDX and PDX mouse models bearing LUADdemonstrated that targeting pan-methylation, glycolysis and even directly against ENO are helpful for the treatments of m^6^A-elevated LUAD. These results highlight the translation significance of the present study.

## Conclusions

The m^6^A-dependent stimulation of glycolysis and tumorigenesis in LUAD is at least partially orchestrated by the upregulation of METTL3, downregulation of ALKBH5, and stimulation of YTHDF1-mediated ENO1 translation (Fig. 8). Blocking this mechanism may be a helpful strategy to treat m^6^A-dependent LUAD.

## Supplementary Information

**Additional file 1.**

**Additional file 2.**

## Data Availability

The data that support the findings of this study are available from corresponding author upon reasonable request.

## Abbreviations

LUAD: lung adenocarcinoma
m^6^A: N6-methyladenosine
METTL3: methyltransferase 3
ALKBH5: alkB homolog 5
ENO1: enolase 1
YTHDF1: YTH N6-methyladenosine RNA binding protein 1
NSCLC: non-small cell lung cancer
METTL14: Methyltransferase 14
WTAP: WT1 associated protein
FTO: FTO alpha-ketoglutarate dependent dioxygenase
HNRNPA2B1: heterogeneous nuclear ribonucleoprotein A2/B1.
IGF2BP: insulin like growth factor 2 mRNA binding protein
ENO: enolase
PEP: phosphoenolpyruvate
2-PGA: 2-phospho-d-glycerate
LLC: Lewis lung cancer cell
qPCR: Quantitative real-time PCR
IB: Immunoblotting
IHC: Immunohistochemical Immunohistochemistry
ELISA: Enzyme-liked Immunsorbent assay
ORF: open reading frame
PAR-CLIP: Photoactivatable ribonucleoside-enhanced crosslinking and immunoprecipitation
RIP: RNA Immunoprecipitation
ECAR: Extracellular acidification rate assay
CDX: cell-derived xenograft
PDX: patient-derived xenograft
NAPSA: napsin A aspartic peptidase
HSPE1: heat shock protein family E member 1
FUCA1: alpha-L-fucosidase 1
ATP5IF1: ATP synthase inhibitory factor subunit 1
TMA: tissue microarray assay
G: guanosine
WT: wild type
Mut: mutant
WBP2: WW domain binding protein2
