# Supplementary material for: The essential roles of m6A RNA modification to stimulate ENO1-dependent glycolysis and tumorigenesis in lung adenocarcinoma
Source: J Exp Clin Cancer Res. 2022 Jan 25;41:36. doi: 10.1186/s13046-021-02200-5 (PMC8788079; doi:10.1186/s13046-021-02200-5)
Supplement: Supplementary file 1 — Additional file 1. [file 13046_2021_2200_MOESM1_ESM.docx]

**Supplementary Figures. 1-7**

**Supplementary Figure. 1**


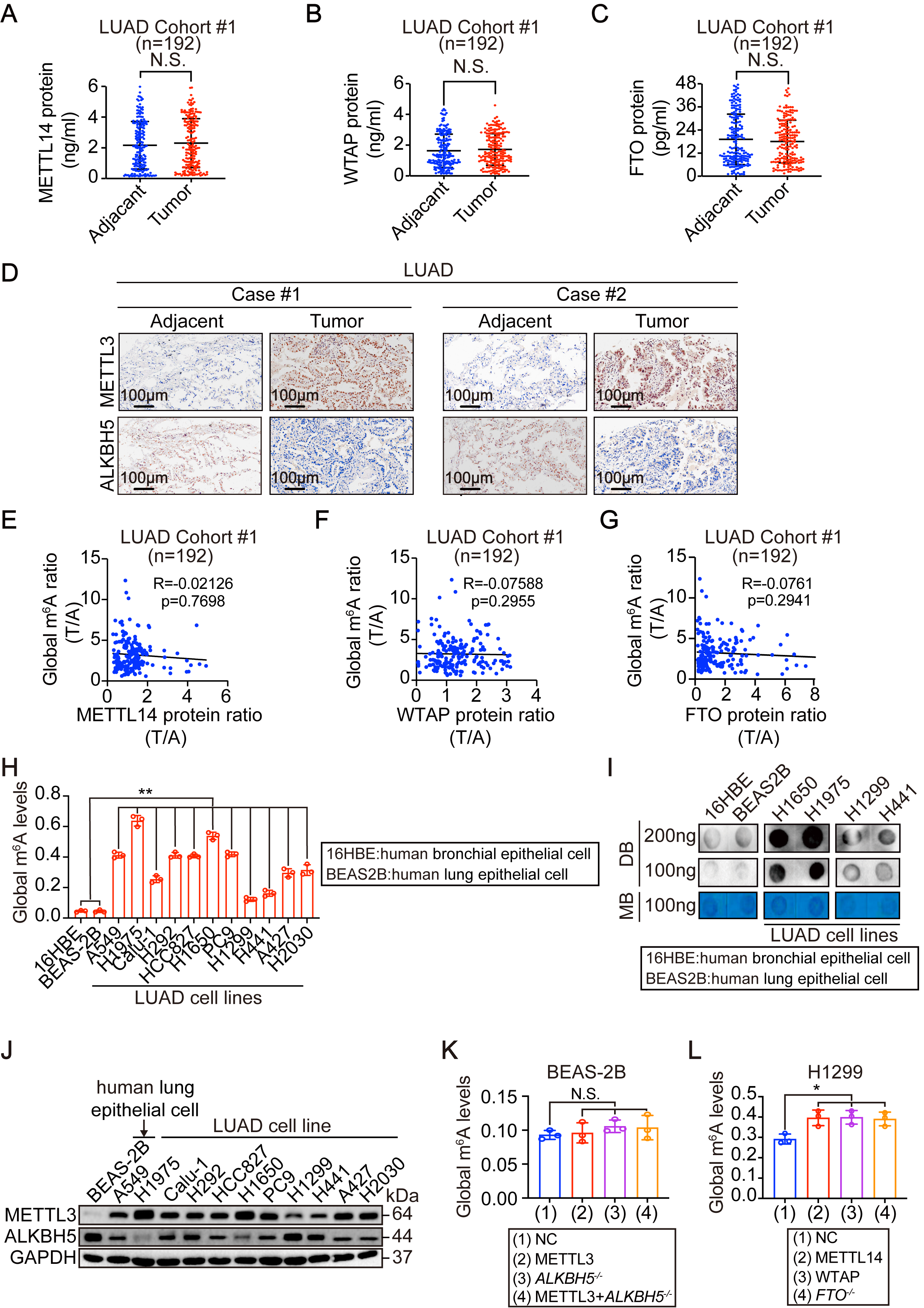


**Supplementary Figure. 1. Supplements to Figure.1.**

(A-C) METTL14 (A), WTAP (B) and FTO (C) protein levels in tumor and its matched-adjacent tissues from LUAD patients, as measured by ELISA.

(D) Representative IHC images of METTL3 and ALKBH5 in tumor and matched-adjacent tissues from 2 randomly chosen LUAD patients. Scale bar, 100 μm.

(E-G) Correlations between global m^6^A level and METTL14 (E), between global m^6^A level and WTAP (F) and between global m^6^A level and FTO (G). The global m^6^A and protein levels were calculated as the ratios between tumor and matched-adjacent tissues.

(H-I) Global m^6^A in indicated established cell lines, as measured by m^6^A methylation assay (H) and dot blot (I), respectively. DB, dot blot, MB, methylene blue.

(J) Representative IB images of METTL3 and ALKBH5 in indicated established cell lines.

(K) Global m^6^A levels in control and BEAS-2B cells with separate or combined METTL3 overexpression and ALKBH5 knockout.

(L) Global m^6^A levels in control and H1299 cells with METTL14 or WTAP overexpression, or FTO knockout.

Statistical analysis was performed using t-test (A-C), spearman rank-correlation analysis (E-G) and one-way ANOVA (H, K, L). Data are presented as means ± SEMs from three independent experiments or indicated samples. **p < 0.01, *p < 0.05 indicates statistical significance and N.S. indicates no significance.

**Supplementary Figure. 2**


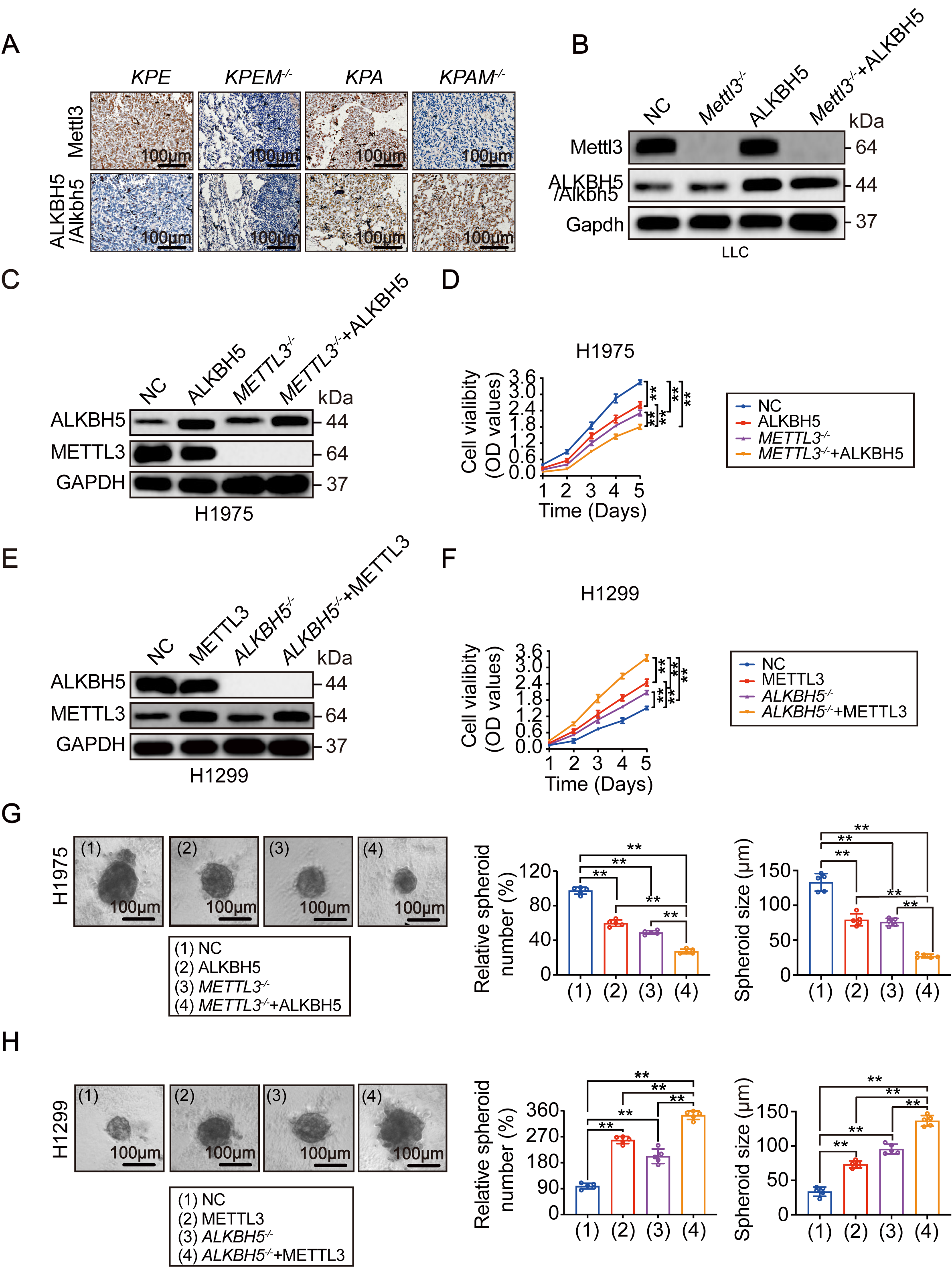


**Supplementary Figure. 2**

**Supplementary Figure. 2. Supplements to Figure. 2.**

(A) Representative IHC images of Mettl3 and ALKBH5/Alkbh5 in spontaneous LUAD from indicated *KP*-based mice. Scale bar, 100 μm.

(B) Representative IB images of Mettl3 and ALKBH5/Alkbh5 of tumors in lung from mice following tail injection of LLC cells with indicated treatment.

(C-D) Representative IB images (C) and cell viability (D) in H1975 cells following separate or combined ALKBH5 overexpression and METTL3 knockout, as indicated.

(E-F) Representative IB images (E) and cell viability (F) in H1299 cells following separate or combined METTL3 overexpression and ALKBH5 knockout.

(G) Representative images, number and size of 3D-spheroids generated by H1975 cells with separate or combined ALKBH5 overexpression and METTL3 knockout. Scale bar, 100 μm.

(H) Representative images, number and size of 3D-spheroids generated by H1299 cells with separate or combined METTL3 overexpression and ALKBH5 knockout. Scale bar, 100 μm.

Statistical analysis was performed using two-way ANOVA (D, F) and one-way ANOVA (G, H). Data are presented as means ± SEMs from three independent experiments. **p < 0.01 indicates statistical significance.

**Supplementary Figure. 3**

**
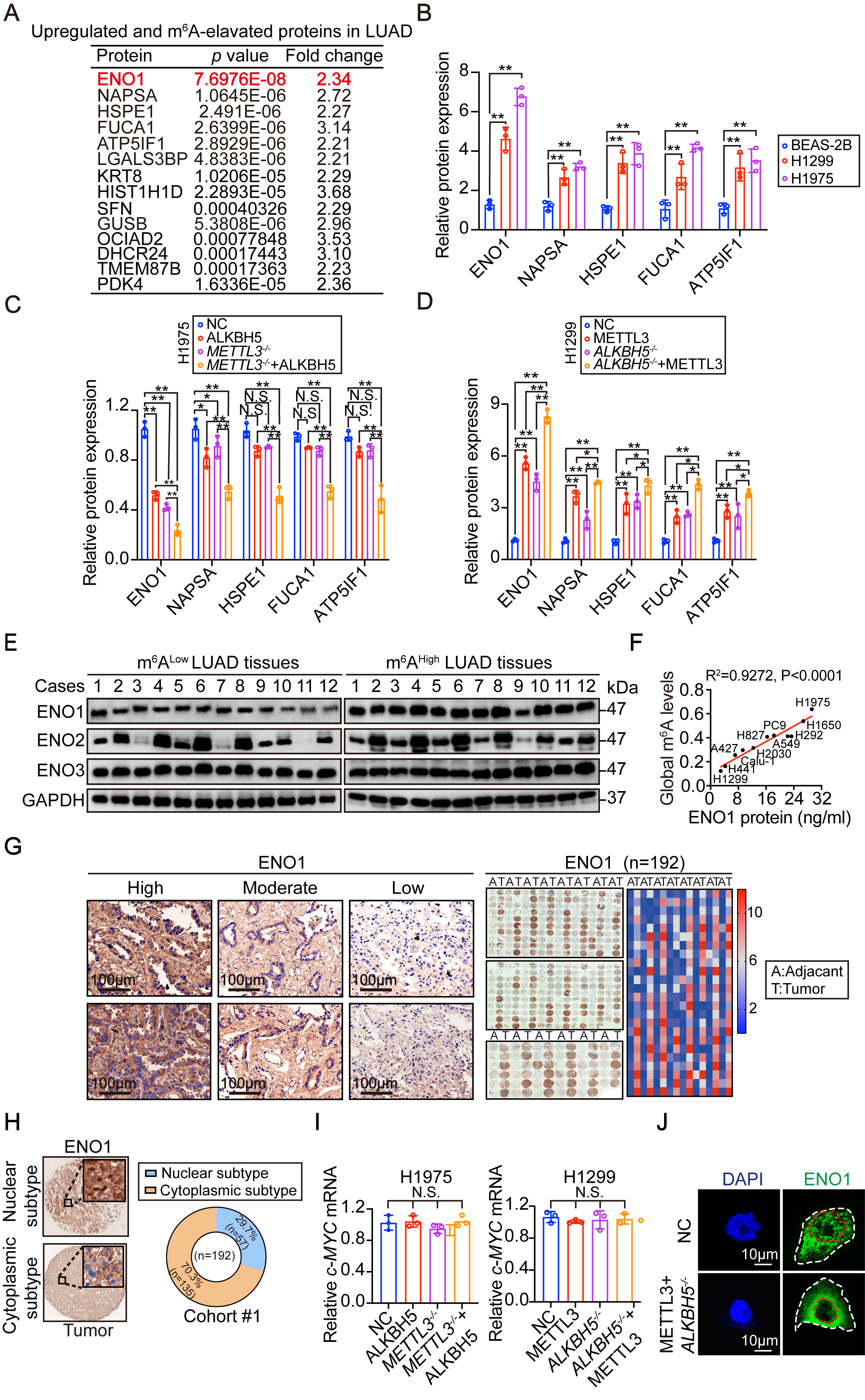
**

**Supplementary Figure. 3**

**Supplementary Figure. 3. Supplements to Figure. 3.**

(A) A list of proteins that predicted to be upregulated in LUAD and elevated by m^6^A, as revealed by proteomics in Figure. 3A.

(B) Comparison of the protein levels of ENO1, NAPSA, HSPE1, FUCA1 and ATP5IF1 in BEAS-2B, H1299 and H1975 cells.

(C-D) The protein levels of ENO1, NAPSA, HSPE1, FUCA1 and ATP5IF1 in H1975 and H1299 cell lines before and after knockout/overexpression of ALKBH5 and METTL3, as indicated.

(E) Measurements of ENO proteins by IB in LUAD tissues with either low or high m^6^A levels.

(F) The correlation between global m^6^A and ENO1 in a serial of LUAD cell lines, as indicated.

(G) Representative IHC images of ENO1 with different expression levels. Also, the heatmaps of TMA are shown. Scale bar, 100 μm.

(H) The representative IHC images of ENO1 with nuclear/cytoplasmic subcellular localization are shown and the percentage of patients with nuclear or cytoplasmic ENO1 subtypes in cohort #1 were also calculated.

(I) *c-MYC* mRNA expression in H1975 and H1299 with different treatments, as measured by RT-qPCR assay.

(J) The representative IF images of ENO1 with changes of subcellular localization in H1299 cells with or without combined MELLT3 overexpression and ALKBH5 knockout. Scale bar, 10 μm.

Statistical analysis was performed using one-way ANOVA (B-D, I) and pearson analysis (F). Data are presented as means ± SEMs from three independent experiments. **p < 0.01, *p<0.05 indicates statistical significance and N.S. indicates no significance.

**Supplementary Figure. 4**

**
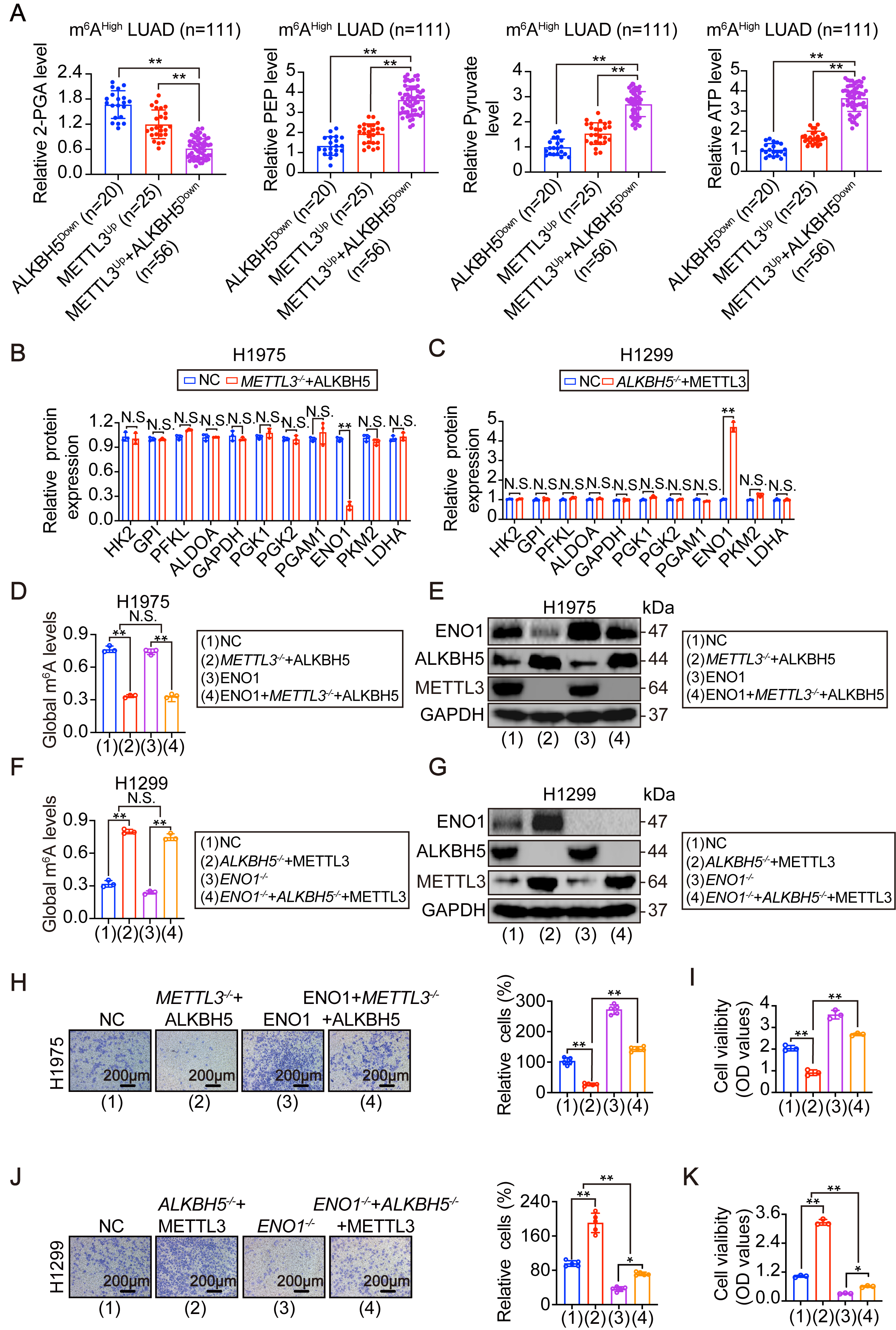
**

**Supplementary Figure. 4**

**Supplementary Figure. 4. Supplements to Figure. 4.**

(A) The 2-PGA, PEP, Pyruvate and ATP in different groups with indicated METTL3 and ALKBH5 expression from LUAD with high global m^6^A levels.

(B-C) The relative expression levels of indicated proteins in H1975 (B) and H1299 cells (C) under treatments, as indicated.

(D-E) Global m^6^A (D), ENO1 (E), ALKBH5 (E) and METTL3 (E) protein expression in H1975 cells with or without combined METTL3 knockout and ALKBH5 overexpression, in the presence or absence of compensation for ENO1.

(F-G) Global m^6^A (F), ENO1 (G), ALKBH5 (G) and METTL3 (G) protein expression in control and *ENO1^-/-^* H1299 cells with or without combined ALKBH5 knockout and METTL3 overexpression.

(H-K) Invasion activity and cell proliferation in H1975 (H-I) and H1299 (J-K) with different treatments, as indicated. The invasion and cell proliferation capacities were measured by transwell- and CellTilter-Glo-based cell viability methods, respectively. Scale bar, 200 μm.

Statistical analysis was performed using t-test (B-C), one-way ANOVA (A, D, F, H-K). Data are presented as means ± SEMs from three independent experiments or indicated samples. **p < 0.01, indicates statistical significance and N.S. indicates no significance.

**Supplementary Figure. 5**


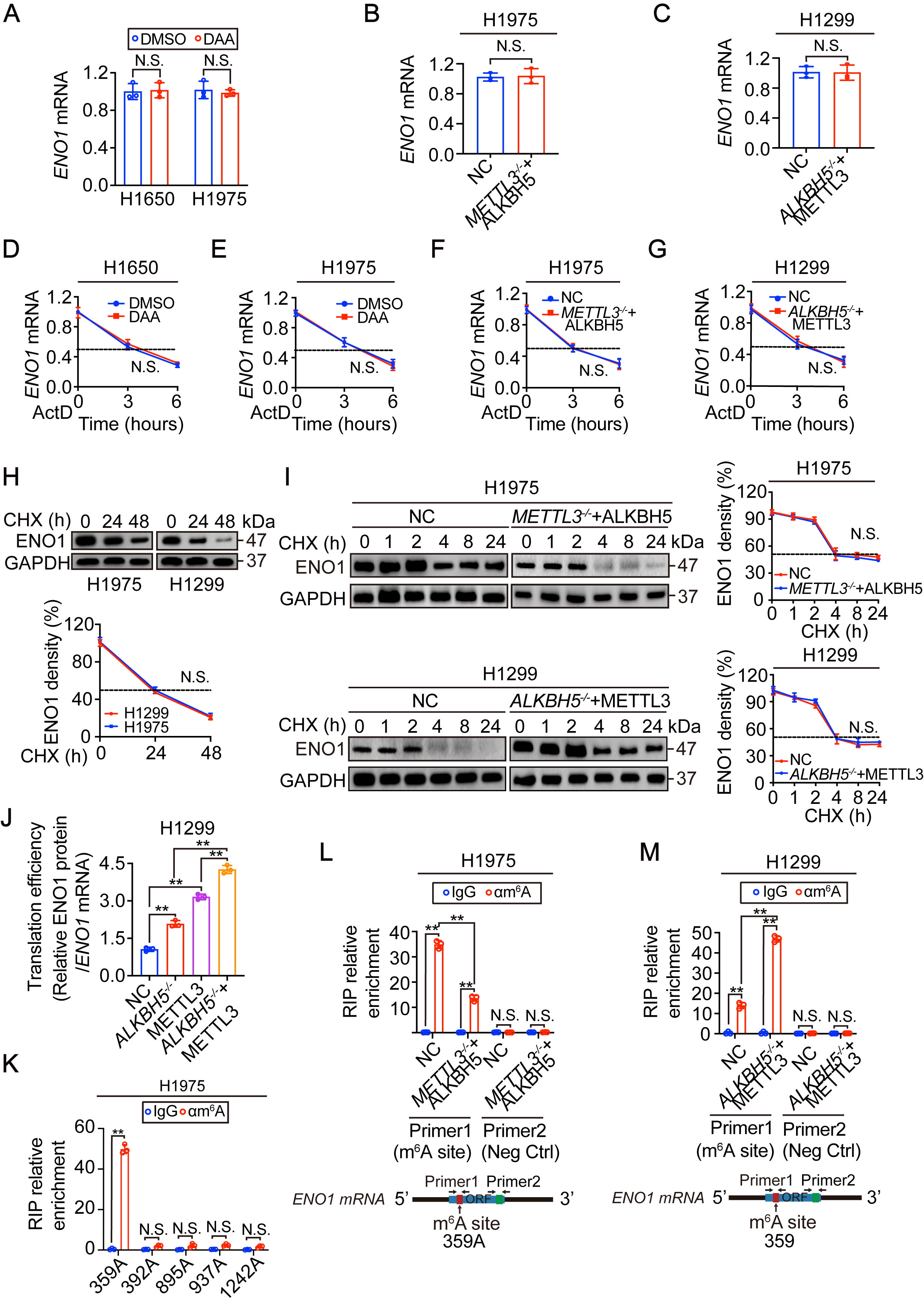


**Supplementary Figure. 5**

**Supplementary Figure. 5. Supplements to Figure. 5.**

(A) *ENO1* mRNA in H1650 and H1975 cells treated with DMSO and DAA (100 μM, 24h).

(B) *ENO1* mRNA in H1975 cells with or without combined METTL3 knockout and ALKBH5 overexpression.

(C) *ENO1* mRNA in H1299 cells with or without combined METTL3 overexpression and ALKBH5 knockout.

(D-E) Actinomycin D chase experiments for *ENO1* mRNA in H1650 (D) and H1975 (E) cells treated with DMSO or DAA (100 μM, 24h).

(F-G) Actinomycin D chase experiments for *ENO1* mRNA in H1975 cells with or without combined METTL3 knockout and ALKBH5 overexpression (F), and in H1299 cells with or without combined METTL3 overexpression and ALKBH5 knockout (G).

(H) CHX chase experiments for detecting ENO1 protein in H1975 and H1299 cells. The ENO1 expression was normalized to that of GAPDH, and the data were also graphed.

(I) ENO1 CHX experiment in H1975 cells with or without combined METTL3 knockout and ALKBH5 overexpression, and in H1299 cells with or without combined ALKBH5 knockout and METTL3 overexpression. The ENO1 expression was normalized to that of GAPDH, and the data were also graphed.

(J) Translation efficiency of ENO1 in H1299 cells with or without separate or combined ALKBH5 knockout and METTL3 overexpression.

(K) RIP using control IgG antibodies and anti-m^6^A antibodies in H1975 cells for detecting m^6^A enrichments at indicated potential m^6^A sites of *ENO1* mRNA.

(L-M) RIP for detecting m^6^A enrichments around the 359A site and at an unrelated region as well in H1975 cells with or without combined METTL3 knockout and ALKBH5 overexpression (L), and in H1299 cells with or without combined ALKBH5 knockout and METTL3 overexpression (M).

Statistical analysis was performed using t-test (A-I, K), one-way ANOVA (J, L, M). Data are presented as means ± SEMs from three independent experiments. **p < 0.01 indicates statistical significance and N.S. indicates no significance.

**Supplementary Figure. 6**


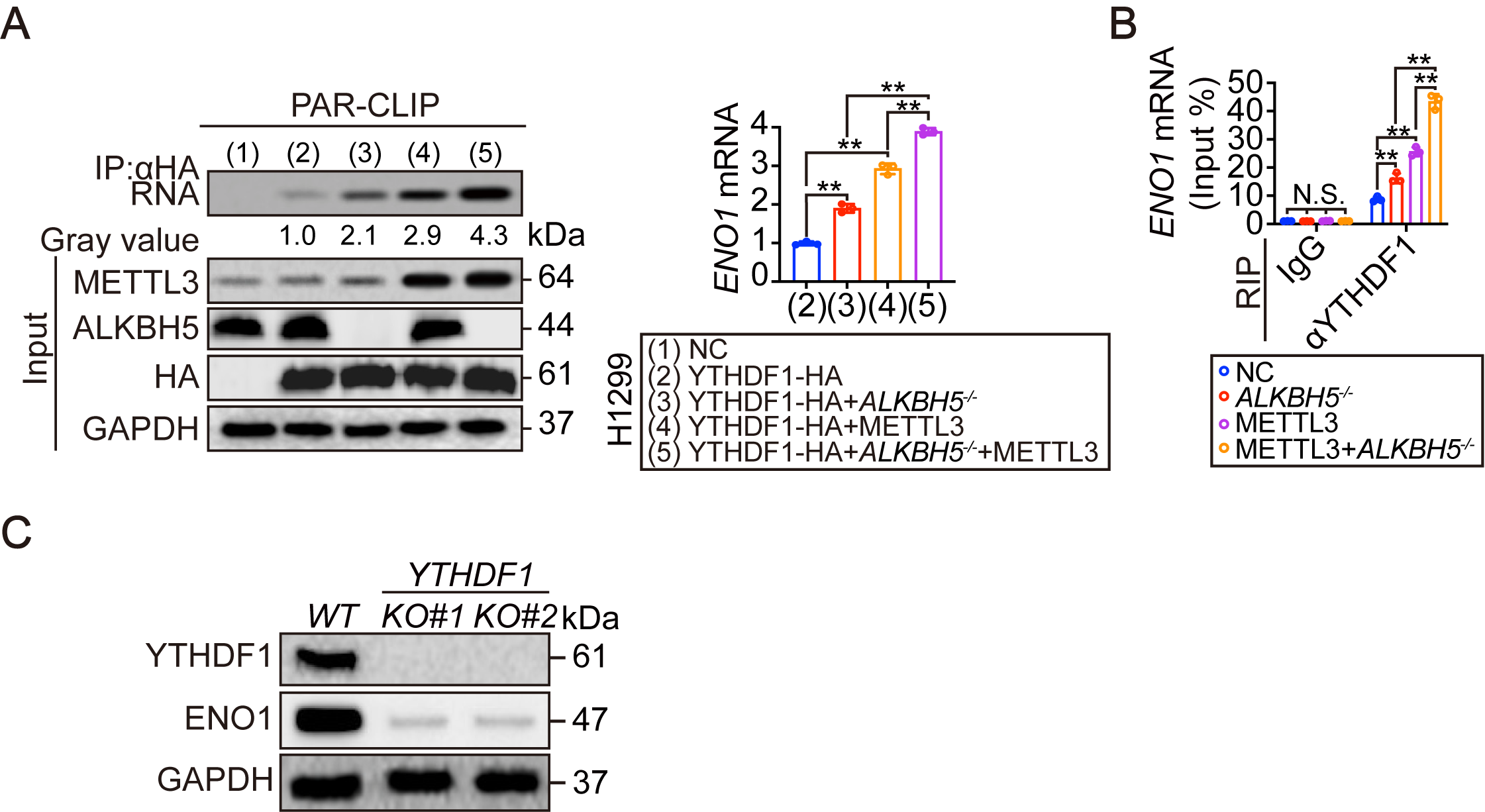


**Supplementary Figure. 6**

**Supplementary Figure. 6. Supplements to Figure. 6.**

(A) YTHDF1 interaction with *ENO1* mRNA, as measured by PAR-CLIP experiment using anti-HA antibodies in H1299 cells expressing HA-tagged YTHDF1, and treated with or without separate or combined ALKBH5 knockout and METTL3 overexpression. RNA labeled with biotin was visualized by the chemiluminescent nucleic acid detection module. *ENO1* mRNA levels in the pulled down products were verified by qPCR.

(B) The association between *ENO1* mRNA and YTHDF1 in H1299 cells with or without separate or combined ALKBH5 knockout and METTL3 overexpression, as measured by RIP experiments using anti-YTHDF1 and control IgG antibodies.

(C) IB of YTHDF1 and ENO1 in *WT* and *YTHDF1-KO* H1299 cells.

Statistical analysis was performed using one-way ANOVA (A, B). Data are presented as means ± SEMs from three independent experiments. **p < 0.01 indicates statistical significance and N.S. indicates no significance.

**Supplementary Figure. 7**


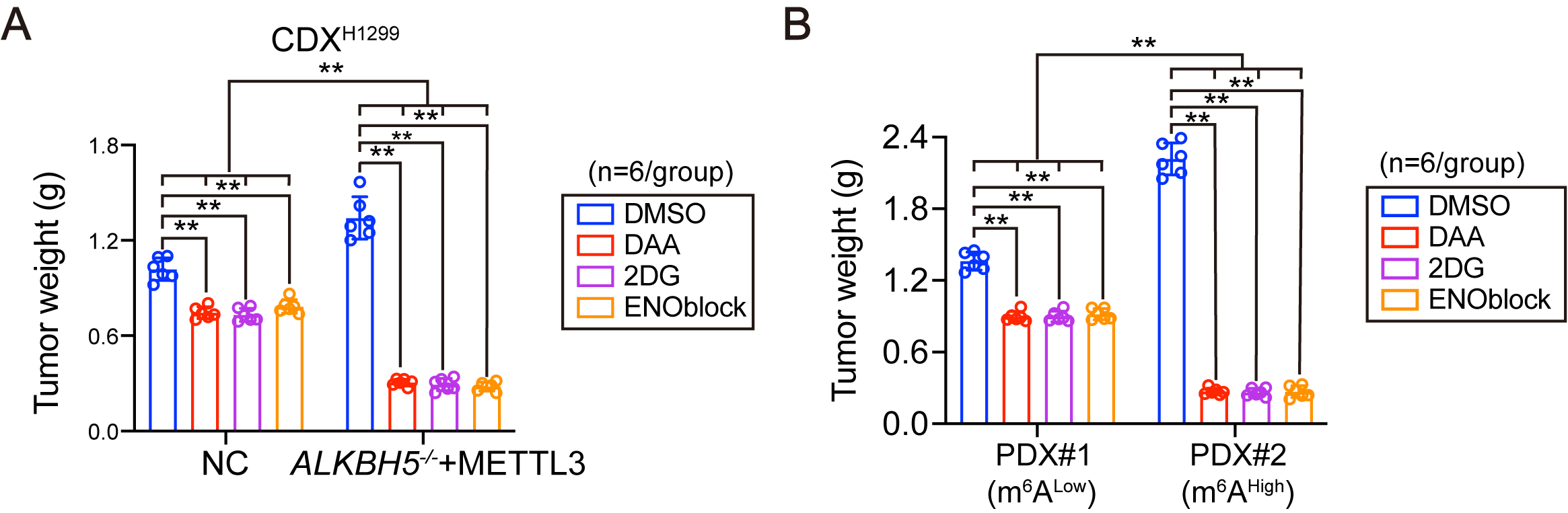


**Supplementary Figure. 7**

**Supplementary Figure. 7. Supplements to Figure. 7.**

(A) Weights of tumors from CDX models that generated by H1299 cells with or without combined ALKBH5 knockout and METTL3 overexpression following being administrated with DMSO, DAA (50mg/kg), 2-DG (1000mg/kg) or ENOblock (20mg/kg).

(B) Weights of tumors from PDX models with low or high levels of global m^6^A following being administrated with DMSO, DAA (50mg/kg), 2-DG (1000mg/kg) or ENOblock (20mg/kg).

Statistical analysis was performed using one-way ANOVA (A, B). Data are presented as means ± SEMs from indicated samples. **p < 0.01 indicates statistical significance.
