# Supplementary material for: The essential roles of m6A RNA modification to stimulate ENO1-dependent glycolysis and tumorigenesis in lung adenocarcinoma
Source: J Exp Clin Cancer Res. 2022 Jan 25;41:36. doi: 10.1186/s13046-021-02200-5 (PMC8788079; doi:10.1186/s13046-021-02200-5)
Supplement: Supplementary file 2 — Additional file 2. [file 13046_2021_2200_MOESM2_ESM.docx]

**Table1. Basal information of patients from cohort#1**

| LUAD patient (Cohort #1, N=192) | | |
| --- | --- | --- |
| Age |  |  |
|  | ≥65 | 98 |
|  | <65 | 94 |
| Gender |  |  |
|  | Male | 100 |
|  | Female | 92 |
| Smoking |  |  |
|  | Yes | 89 |
|  | No | 103 |
| Stage |  |  |
|  | I | 65 |
|  | II | 67 |
|  | III | 60 |
| Total |  | N=192 |

**Table2. Basal information of patients from cohort#2**

| LUAD patient (Cohort #2, N=186) | | |
| --- | --- | --- |
| Age |  |  |
|  | ≥65 | 74 |
|  | <65 | 112 |
| Gender |  |  |
|  | Male | 104 |
|  | Female | 82 |
| Stage |  |  |
|  | I | 16 |
|  | II | 108 |
|  | III | 62 |
| Total |  | N=186 |
|  |  |  |

**Table3. Basal information of patients from cohort#3**

| LUAD patient (Cohort #3, N=60) | | |
| --- | --- | --- |
| Age |  |  |
|  | ≥65 | 34 |
|  | <65 | 26 |
| Gender |  |  |
|  | Male | 31 |
|  | Female | 29 |
| Smoking |  |  |
|  | Yes | 32 |
|  | No | 28 |
| Stage |  |  |
|  | I | 20 |
|  | II | 20 |
|  | III | 20 |
| Total |  | N=60 |

**Table4. The sequence of sgRNA and cloning primers**

| **Name** | **Sequence** |
| --- | --- |
| h-sg-ALKBH5 | GATCAACGACTACCAGCCCGG |
| h-sg-FTO | GTTCTGGCGTGCAGTGAGCG |
| h-sg-METTL3 | ATATCACAACAGATCCACTG |
| m-sg-Mettl3 | ACGCCGTTTCTGCCCTGCGA |
| h-sg-ENO1 | GTACCGCCACATCGCTGACT |
| h-sg#1-YTHDF1 | GAACAGCCTGGAGCAGGGCA |
| h-sg#2-YTHDF1 | GGGATGAACAGCCTGGAGCA |
| h-YTHDF1-HA-F | GACTGCTAGCATGTCGGCCACCAGCGTGGAC |
| h-YTHDF1-HA-R | GACTGGATCCTCATTGTTTGTTTCGACTCTG |
| h-YTHDF1^ΔYTH^-HA-F1 | AATCTGAAAAGCGGGAGTTCCTACAAGCACACA |
| h-YTHDF1^ΔYTH^-HA-R1 | GTGCTTGTAGGAACTCCCGCTTTTCAGATTCCA |

**Table5. The primer sequence of qPCR, RIP and PAR-CLIP-qPCR**

| **Name** | **Sequence (Forword 5'-3')** |
| --- | --- |
| h-ENO1-qPCR-F | TCAAGGACTACCCCGTGGTG |
| h-ENO1-qPCR-R | AGGCAGTTGCAGGACTTCTCGTT |
| h-c-MYC-qPCR-F | CGTCTCCACACATCAGCACAA |
| h-c-MYC-qPCR-R | CTCTTGGCAGCAGGATAGTCCTT |
| h-ENO1-RIP-qPCR-359 (primer1)-F | GGTATCTATGAGGCCCTA |
| h-ENO1-RIP-qPCR-359 (primer1)-R | TCAATCTTCTCTTGTTCT |
| h-ENO1-RIP-qPCR-392-F | GAACGTCACAGAACAAGA |
| h-ENO1-RIP-qPCR-392-R | GGTACAGGGGGACCCCCT |
| h-ENO1-RIP-qPCR-895-F | GCTACACTGATAAGGTGG |
| h-ENO1-RIP-qPCR-895-R | GATGAAGGACTTGTACAG |
| h-ENO1-RIP-qPCR-937-F | CATCTCGCCTGACCAGCT |
| h-ENO1-RIP-qPCR-937-R | GATCATCCCCCACTACCT |
| h-ENO1-RIP-qPCR-1242-F | CTCTTCAGGCGTGCAAGC |
| h-ENO1-RIP-qPCR-1242-R | CTCAGATCGGCAAGGGGC |
| h-ENO1-RIP-qPCR-primer2-F | GTTGTGGGGCTGTGCACT |
| h-ENO1-RIP-qPCR-primer2-R | CTTGGCCAAGGGGTTTCT |
| h-ENO1-RIP/PAR-CLIP-qPCR-F | GGTATCTATGAGGCCCTA |
| h-ENO1-RIP/PAR-CLIP-qPCR-R | TCAATCTTCTCTTGTTCT |

**Table6. The list of antibodies for IB, IF and IHC assay**

| Name | Sources |
| --- | --- |
| METTL3 | Abcam, ab195352 |
| ALKBH5 | Abcam, ab195377 |
| GAPDH | Abcam, ab#181602 |
| m^6^A | Synaptic Systems, #202003 |
| ENO1 | Abcam, ab#227978 |
| ENO2 | Abcam, ab#180943 |
| ENO3 | Abcam, ab#157474 |
| NAPSA | Abcam, ab#133249 |
| HSPE1 | ImmunoWay, YT#2248 |
| FUCA1 | Abcam, ab#181357 |
| ATP5IF1 | Cell Signaling Technology, #8528S |
| YTHDC1 | Abcam, ab220159 |
| YTHDC2 | Abcam, ab176846 |
| YTHDF1 | Abcam, ab#220162 |
| YTHDF2 | Abcam, ab#220163 |
| YTHDF3 | Abcam, ab#220161 |
| hnRNPA2B1 | Abcam, ab#31645 |
| IGF2BP1 | Abcam, ab#ab184305 |
| IGF2BP2 | Abcam, ab#ab129071 |
| IGF2BP3 | Abcam, ab#179807 |
| HA | Abcam, ab#1424 |
| GAPDH | Cell Signaling Technology, #5174, #97166 |
| HK2 | Abcam, ab209847 |
| GPI | CST, 94068T |
| PFKL | Abcam, ab97443 |
| ALDOA | CST, 3188S |
| PGK1 | Abcam, ab199438 |
| PGK2 | Abcam, ab183031 |
| PGAM1 | Abcam, ab129191 |
| PKM2 | CST, 4053T |
| LDHA | CST, 2012S |
|  |  |
